# LSARE-Net: Target-Guided Augmentation and Lightweight Edge Detection for Real-Time Behavior Monitoring of Captive Juvenile Sika Deer

**DOI:** 10.3390/ani16193094

**Published:** 2026-10-01

**Authors:** Xipeng Li, He Pan, Adnan Mahmood, Xin Wang, Tong Yu, Yuncheng Zhao

**Affiliations:** 1 College of Information Technology, Jilin Agricultural University, Changchun 130118, China; 20241269@mails.jlau.edu.cn (X.L.); yutong@mails.jlau.edu.cn (T.Y.); 20241237@mails.jlau.edu.cn (Y.Z.); 2 Information Center, Jilin Agricultural University, Changchun 130118, China; panhe@jlau.edu.cn; 3 School of Computing, Macquarie University, Sydney, NSW 2109, Australia; adnan.mahmood@mq.edu.au

**Keywords:** juvenile sika deer, animal behavior monitoring, precision livestock farming, computer vision, lightweight object detection, edge computing

## Abstract

Real-time machine-vision monitoring of group-housed juvenile sika deer is challenging because targets are often small, densely distributed and partially occluded, while edge deployment requires compact models. This study developed a lightweight system for non-contact behavior recognition under commercial farm-pen conditions. A fixed-camera dataset was constructed with five annotated categories: resting, standing, feeding, social and abnormal. The dataset was limited to one month of recordings from one fixed camera monitoring six juvenile sika deer in a single pen at one farm in Jilin Province, China. To reduce background interference during training, a Multi-target Bionic Foveal Augmentation (MBFA) strategy was proposed to generate target-guided, spatially nonuniform degradation based on the distribution of deer instances, without introducing additional inference cost. A Lightweight Selective Alignment Reorganization and Efficient Detection Network (LSARE-Net) was then designed to enhance local behavioral cues, contextual feature representation, and cross-scale fusion. On the test set, LSARE-Net achieved 91.26% recall, 93.47% mAP@0.5 and 80.24% mAP@0.5:0.95 with 0.70 M parameters and a 1.7 MB model size. After TensorRT conversion, it reached 62.64 FPS on an NVIDIA Jetson AGX Orin. These results demonstrate the feasibility of compact frame-level behavior detection under the evaluated pen conditions. The accompanying prototype organizes detections into behavioral summaries and abnormal-cue records for manual review.

## Simple Summary

While continuous monitoring can reveal changes in deer behavior, manually reviewing surveillance footage is both time-consuming and labor-intensive. To address this, we developed a camera-based system to detect five behavioral categories in group-housed juvenile sika deer: resting, standing, feeding, social interaction, and abnormal recumbency. The system was developed using a dataset of 5600 images from six deer housed in one pen and incorporated a compact model suitable for on-site deployment. The model contained 0.70 million parameters and achieved 62.64 frames per second on an NVIDIA Jetson AGX Orin device (NVIDIA Corporation, Santa Clara, CA, USA). The monitoring prototype organized detections into behavioral summaries and abnormal-cue records for human review.

## 1. Introduction

Sika deer are economically important farmed cervids in China, with approximately 852,000 animals raised in Jilin Province alone by the end of 2023, accounting for more than half of the country’s total farmed sika deer population [1]. Velvet antler and related products constitute major sources of economic value for the Chinese deer industry [2]. As intensive captive farming expands, sika deer production places greater demands on herd management, health monitoring, and standardized husbandry while also making individual behavioral observation and health-status monitoring more difficult [3]. Continuous behavioral monitoring is particularly important for juvenile sika deer because standing and suckling behaviors and mother–young interactions are established during the early postnatal period [4]. Monitoring changes in these behaviors may help identify individuals requiring closer health observation and inform feeding and husbandry decisions relevant to growth and development. However, behavioral changes in potential concern are often subtle, intermittent and time-varying, making them difficult to capture through discontinuous and subjective manual inspection [5]. Automated monitoring of behavioral responses has therefore become an important non-invasive approach to continuous behavioral data acquisition in precision livestock farming [6].

Wearable sensors have been widely used to monitor livestock and poultry behavior and health status, including activity, feeding, rumination and body-temperature-related indicators [7,8,9]. Nevertheless, these devices may entail installation and maintenance costs, limited battery life, a risk of device damage, and potential interference with natural animal movement. These limitations are particularly relevant in juvenile deer farming systems characterized by frequent group movement and close contact among individuals [10]. Therefore, non-contact vision-based monitoring provides a promising alternative for continuous behavioral observation under practical farming conditions.

Deep learning-based vision methods have been increasingly applied to livestock and poultry behavior recognition because they can detect targets and classify behavioral states from video or image data without physical contact [11]. For example, computer-vision models have been used to analyze piglet feeding behavior and aggression-related dynamics from video recordings, supporting the value of vision-based behavioral indicators in farm management [12]. Two-stage detection frameworks, such as Faster R-CNN, have shown favorable localization accuracy in animal posture and feeding-behavior recognition, but their region-proposal and multi-stage regression procedures increase computational complexity and inference latency [13,14]. In contrast, YOLO-based single-stage detectors have been widely used in livestock behavior recognition because of their end-to-end prediction structure, fast inference, and ease of deployment [15,16,17].

Recent studies have attempted to improve recognition robustness through illumination-adaptive design, local motion modeling and spatiotemporal feature fusion [18,19,20]. Meanwhile, edge AI systems have been developed for real-time livestock and poultry monitoring on platforms such as Jetson Orin NX and Jetson AGX Orin, demonstrating the potential of local inference in practical farm environments [21,22,23]. However, most existing deployment-oriented studies focus on common livestock and poultry species, such as cattle, pigs, sheep and chickens. Edge-based behavior monitoring for captive juvenile sika deer remains insufficiently explored.

Visual recognition studies on sika deer have mainly focused on posture recognition, key-part detection, body-part detection, behavior recognition and individual identification [24,25,26,27]. These studies provide a useful foundation for non-contact deer monitoring. However, existing work has paid limited attention to target-instance-level multi-class behavior detection, abnormal cue recording and edge-based monitoring in real-world captive juvenile-deer settings. In pen-monitoring videos, juvenile deer often appear as small and densely distributed targets, with frequent partial occlusion and strong background interference caused by long-distance imaging, illumination changes, ground textures and enclosure structures. These conditions call for methods that maintain reliable detection while remaining computationally efficient for edge deployment.

Therefore, this study proposes a lightweight machine-vision system for real-time behavioral monitoring of captive juvenile sika deer under complex enclosure conditions. The study was designed to test whether target-guided training augmentation and a lightweight aligned detection network could improve behavior detection performance under complex pen conditions while maintaining efficient edge-device inference. Given that the data were obtained from one fixed camera monitoring six juvenile sika deer in a single pen at one farm in Jilin Province over one month, the present study focuses on technical feasibility under the recorded conditions. The main contributions of this study are as follows:

- An annotated behavior detection dataset for captive juvenile sika deer was constructed under real pen conditions. The dataset covers five annotated behavioral categories, namely resting, standing, feeding, social, and abnormal, with corresponding operational annotation criteria.
- A lightweight vision method suitable for edge deployment was developed for complex pen environments, combining MBFA-based training augmentation with the LSARE-Net detector. The method improves the detection of small, occluded, and distant targets while reducing parameter count and model size.
- Edge-deployment evaluation was performed on the NVIDIA Jetson AGX Orin platform. In addition, a monitoring workflow was developed to convert frame-level detections into behavioral proportions, abnormal-presence duration, and event records for routine farm-management review.

## 2. Materials and Methods

### 2.1. Rationale for Dedicated Dataset Construction

For captive juvenile sika deer behavior detection, a task-specific dataset is necessary for model development and evaluation. To determine whether existing data resources could support this task, publicly available deer-related image datasets were reviewed and compared, as shown in Table 1. Existing datasets are mainly designed for deer object detection, multi-species camera-trap recognition, or wildlife monitoring tasks. Although these resources can support deer localization or species identification, they differ substantially from captive juvenile sika deer behavior detection in terms of acquisition scenario, annotation granularity, and task objective. Existing public datasets do not provide target-instance-level behavior annotations under real captive enclosure-surveillance conditions. They also do not cover the five behavior categories considered in this study, namely resting, standing, feeding, social, and abnormal. Therefore, these datasets cannot directly support the development and evaluation of a behavior detection model for captive juvenile sika deer. Given these limitations, a dedicated dataset was constructed from non-contact surveillance videos collected under real farm conditions.

### 2.2. Study Site and Video Acquisition

**Figure 1:**
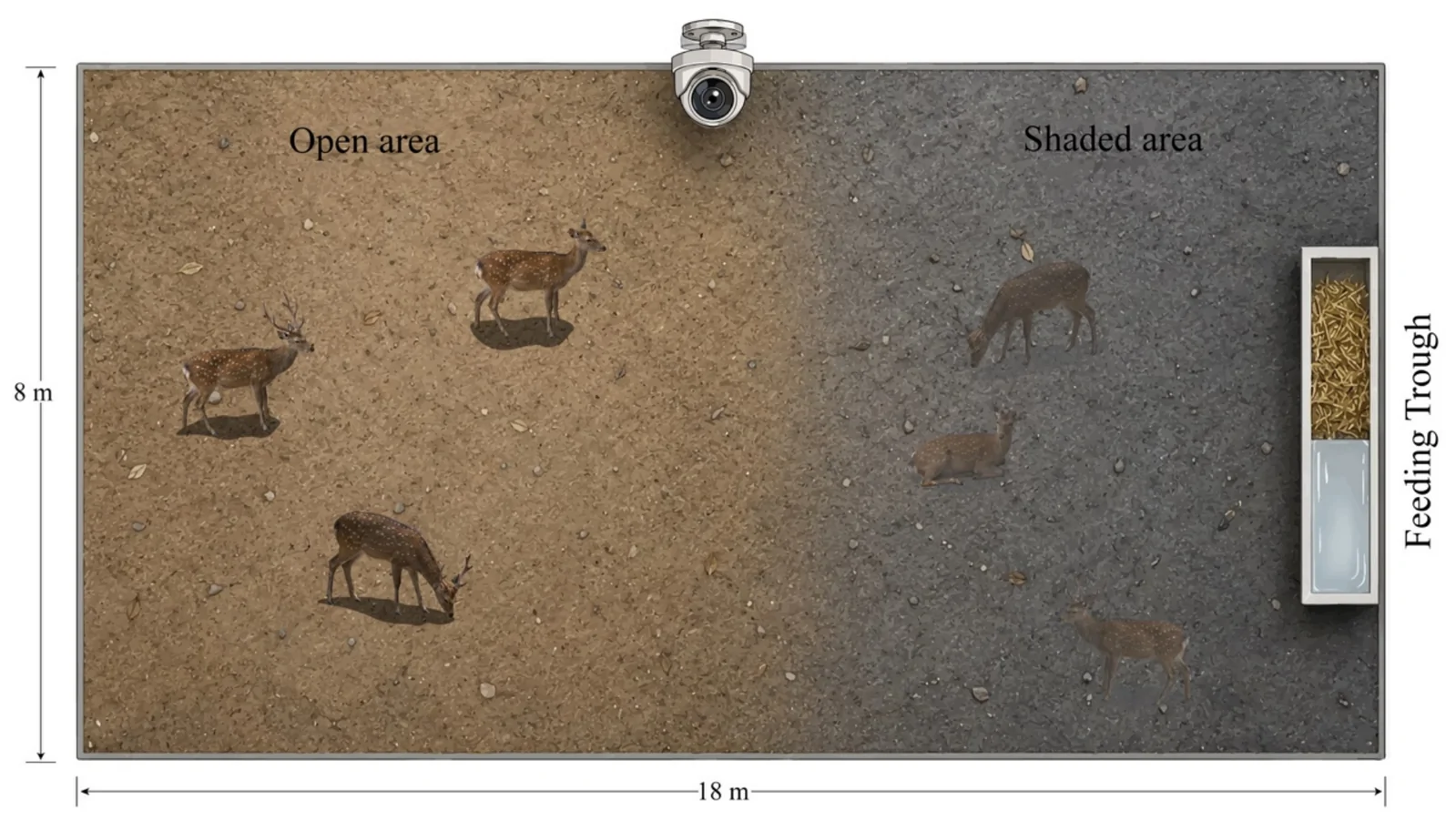
Layout of the juvenile sika deer pen showing the open activity area, sheltered area, feeding trough, and fixed camera location.

The study was conducted in a semi-open sika deer pen at Nanshan Lüke Deer Farm, located in Lujia Village, Xiaogushan Town, Yitong Manchu Autonomous County, Siping City, Jilin Province, China. The pen measured 18 m × 8 m with a height of 3.4 m and consisted of an open activity area, a sheltered area, and feeding and drinking zones. Six juvenile sika deer (three males and three females), approximately 15–35 days old at the start of recording, were housed in the pen and managed according to the farm’s routine husbandry schedule. The term “juvenile” in this study refers to this early postnatal cohort (less than 3 months of age). Body weight was estimated to range from 10 to 15 kg; exact individual ages and measured individual body weights were not available. Video was recorded non-invasively using a fixed camera. Recording did not interfere with routine feeding, watering, cleaning, or group-management procedures. To ensure stable coverage of the main activity area, the surveillance camera was mounted on the top beam of the pen and directed toward the center of the activity area. Video data were collected using a Hikvision Q4P dual-lens 4G network camera, model HK-Q4PADM-GL/L (Hangzhou Hikvision Digital Technology Co., Ltd., Hangzhou, China). Each channel provided a resolution of 2960 × 1664 pixels, supported H.265 video encoding, and was equipped with white-light and infrared supplementary illumination. Video recordings were collected from 1 July 2025 to 30 July 2025. The pen layout and camera installation position are shown in Figure 1.

### 2.3. Image Dataset Construction and Annotation Protocol

Surveillance videos collected under farm conditions were manually reviewed to identify and record periods containing the five target behaviors according to the operational criteria in Table 2. A differential frame-sampling strategy was adopted to retain behavioral examples while reducing inter-frame redundancy. Periods containing social interactions or abnormal recumbency were sampled at 1 frame/s, including periods when resting, standing, or feeding occurred simultaneously; other selected periods containing resting, standing, or feeding were sampled at 1 frame/60 s. These sampling intervals were used only for dataset construction and did not constrain the frame-processing rate during deployment.

This strategy increased the representation of periods containing social interactions or abnormal recumbency, including other behaviors visible in the same frames; consequently, dataset category frequencies should not be interpreted as estimates of natural behavioral prevalence or time allocation. Performance metrics reflect this selected sampling distribution and may differ from those obtained during continuous monitoring at natural behavioral frequencies. All models were evaluated on the same sampled test set, but the magnitude and direction of the sampling effect on performance estimates were not quantified.

**Figure 2:**
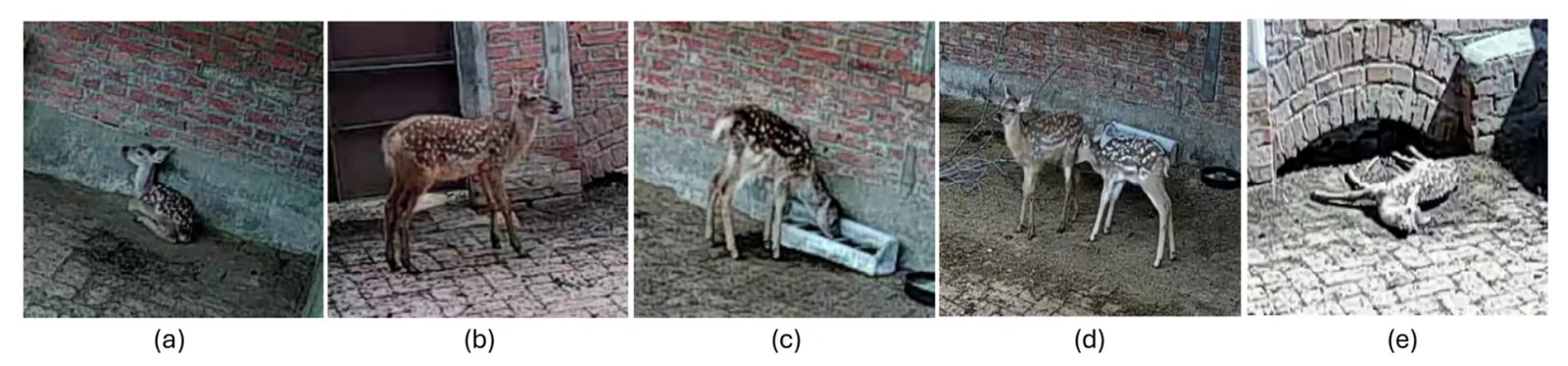
Representative surveillance-frame examples of five behavioral states in juvenile sika deer: (**a**) resting, (**b**) standing, (**c**) feeding, (**d**) social, and (**e**) abnormal recumbency. Normal resting in (**a**) is characterized by naturally folded limbs and a supported head–neck posture, whereas abnormal recumbency in (**e**) shows splayed limbs and a lowered head–neck posture lacking normal support, consistent with the annotation criteria in Table 2.

After frame extraction, images with severe blur, incomplete field of view, or invalid surveillance content were removed. Deer instances in the retained images were annotated using five visually observable categories, resting, standing, feeding, social, and abnormal, with abnormal restricted to visually observable abnormal recumbency. Table 2 provides the category definitions and criteria for distinguishing potentially confusable behaviors, while Figure 2 presents representative surveillance-frame examples.

Bounding-box annotation of juvenile deer behavior targets was performed using X-AnyLabeling Version 3.0.0 [28]. Before annotation, the annotator consulted deer-farm personnel about the behavioral characteristics of juvenile sika deer and used their input to develop the operational annotation criteria presented in Table 2. All annotations were completed by a single annotator, who also reviewed and corrected the bounding boxes and behavioral labels. Behavioral labels were assigned through manual inspection of the original images and source videos, without reference to model predictions. Annotation and self-review were completed before dataset partitioning, so training, validation, and test assignments were not available during annotation. When normal resting was difficult to distinguish from abnormal recumbency in a single image, the annotator reviewed the corresponding source video to assess the temporal context surrounding the frame. The abnormal category is limited to visually observable abnormal recumbency for event recording. Normal recumbency and cases attributable solely to brief postural adjustments, turning, or occlusion-related incomplete limb visibility are excluded from this category.

### 2.4. Dataset Split and Augmentation Strategy

**Figure 3:**
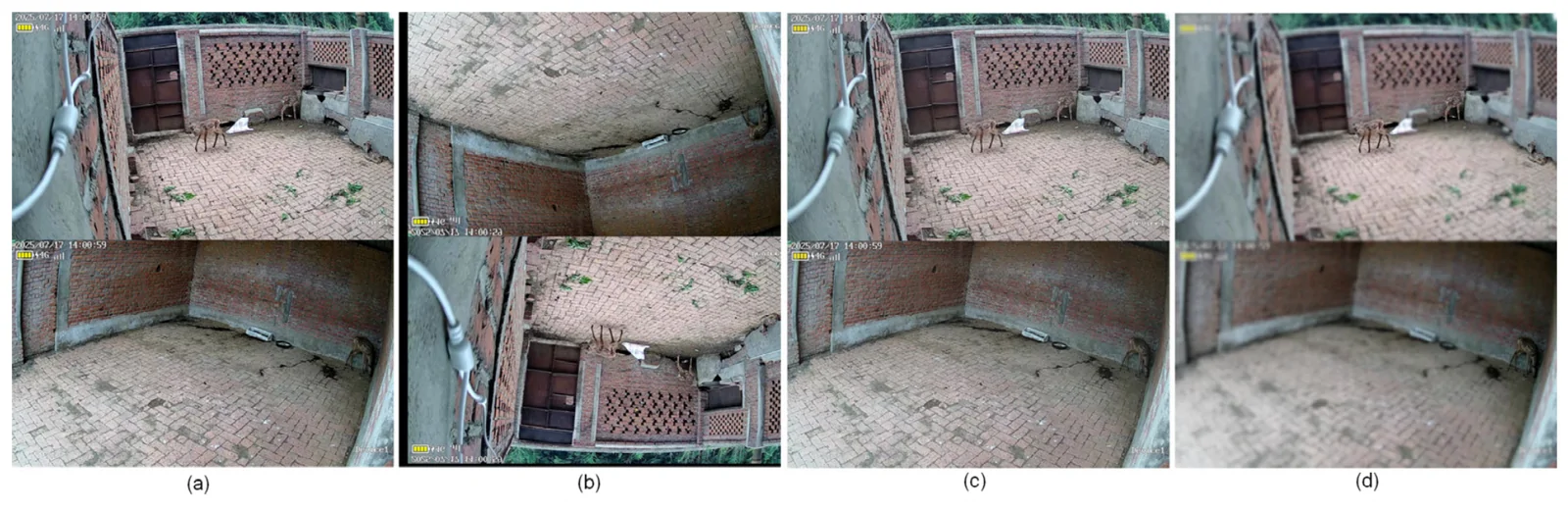
Examples of data augmentation results for juvenile sika deer behavior images: (**a**) original image; (**b**) geometrically augmented image; (**c**) appearance-augmented image; and (**d**) image augmented using multi-target bionic foveal augmentation.

The 5600 original images were randomly partitioned at the image level, without grouping by source video or recording date, into training, validation, and test subsets containing 2063, 2358, and 1179 images, respectively. Augmentation was applied only after partitioning and only to the training subset, increasing its size fourfold from 2063 to 8252 images, while the validation and test subsets remained unaugmented. The resulting approximately 7:2:1 ratio describes post-augmentation image counts rather than the partition proportions of the original images. Because the original training images did not fully represent the expected variation in illumination, motion blur, background clutter, target scale, and day–night appearance, data augmentation was used to increase imaging diversity. Geometric augmentation included rotation, translation, scaling, and flipping; appearance augmentation included blur, noise injection, and contrast perturbation; and multi-target bionic foveal augmentation applied spatially varying blur and saturation attenuation according to the locations of annotated targets. The original images and examples of different augmentation results are shown in Figure 3. Because a single image may contain multiple juvenile deer targets, the number of images differs from the number of target instances. Table 3 reports class-specific image and target-instance counts for the training set before and after augmentation, including the original images in the latter, and for the unaugmented validation and test sets. Each image is counted once for every category it contains, so class-specific image counts may sum to more than the total number of distinct images, whereas instance counts represent the numbers of annotated bounding boxes.

### 2.5. Multi-Target Bionic Foveal Augmentation

**Figure 4:**
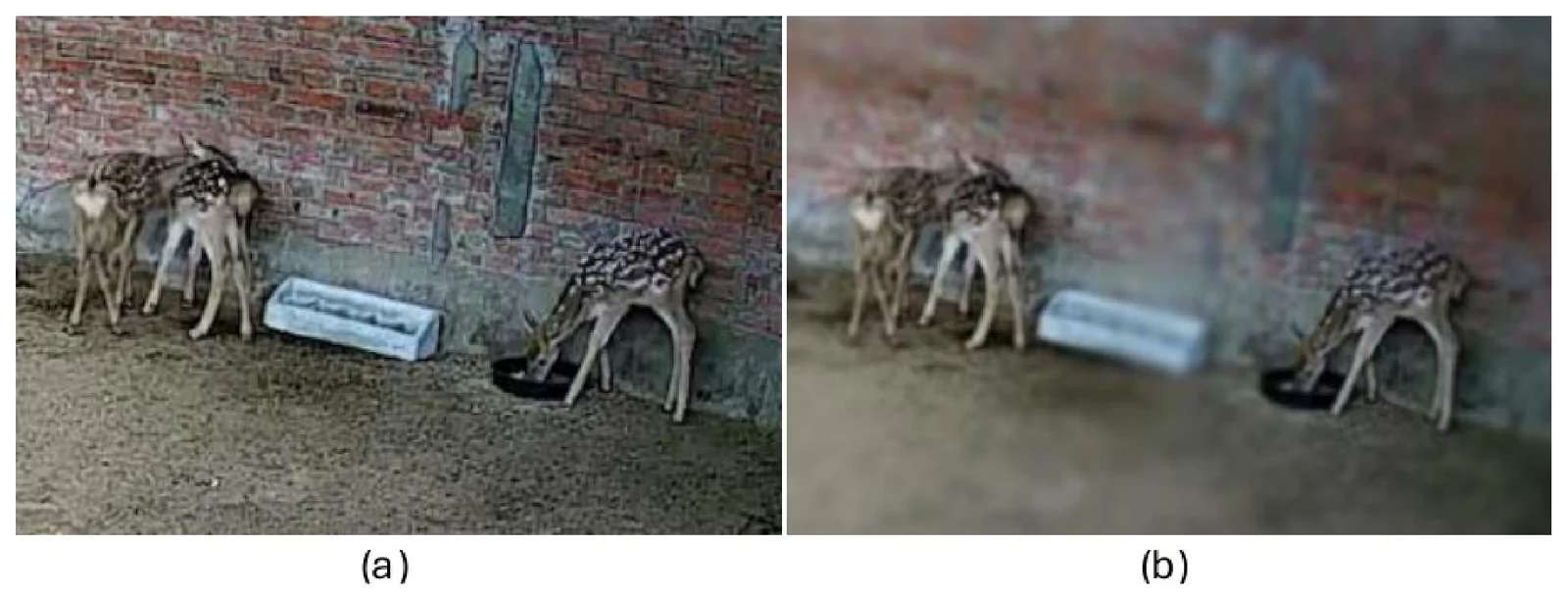
Schematic illustration of multi-target bionic foveal augmentation for juvenile sika deer behavior images: (**a**) original image; and (**b**) image after MBFA.

Surveillance images often contain multiple juvenile deer, and background textures can interfere with the extraction of behavior-related features. Inspired by the foveated imaging mechanism of the human visual system, R-Blur applies spatially variant blur around a single fixation point, together with peripheral saturation attenuation, to guide the model toward core information within the clear region [29]. However, this method is mainly designed for single-subject or single-fixation scenarios. When applied to multi-object detection tasks, its clear region may fail to cover multiple spatially separated targets simultaneously, which may reduce the amount of useful texture and contour information retained for targets far from the fixation point. To address this limitation, this study proposes a Multi-target Bionic Foveal Augmentation (MBFA) method. Based on the spatial distribution of targets within the same image, MBFA constructs multiple dynamic target clusters, preserving more complete texture, edge, and contour information in target regions and their neighboring areas. Meanwhile, progressive blur and saturation attenuation are applied to background regions far from the targets, thereby reducing the interference of complex pen backgrounds during model training. MBFA generates augmented images only during the training stage based on the ground-truth bounding boxes of the current sample. During inference, the original image is directly used as input, without requiring additional annotations, fixation priors, or post-processing modules. Therefore, MBFA does not introduce extra computational overhead during model deployment. The augmentation effect of MBFA is shown in Figure 4.

First, target clusters were constructed according to the overlap relationships among bounding boxes. For any two target bounding boxes, *B_i_* and *B_j_*, their intersection over union (IoU) is defined as:

(1) $$\mathrm{IoU}(B_{i},B_{j})=\frac{\left|B_{i}\cap B_{j}\right|}{|B_{i}\cup B_{j}|+\varepsilon }$$

Bounding boxes were grouped into target clusters through connected components defined by pairwise IoU > *τ*, with *τ* = 0.3; boxes without qualifying connections formed individual clusters. For valid, nondegenerate boxes, the union area is strictly positive, so *ε* is not mathematically required to define IoU. Nevertheless, the implementation added *ε* = 10^−6^ to the union-area denominator as a defensive numerical safeguard. For each cluster, the foveal center was placed at the center of the common intersection of its boxes when that intersection had positive area; otherwise, the center of the smallest enclosing rectangle was used. Let *W* and *H* denote the width and height of this enclosing rectangle. The corresponding foveal radius is defined as:

(2) $$r_{k}=\max\left(\lambda \cdot \frac{1}{2}\sqrt{W_{k}^{2}+H_{k}^{2}},1\right)$$

where *λ* is the foveal-region enlargement coefficient and is set to 1.5. This design allows the foveal region to cover not only the target bounding box itself but also the surrounding local contextual information relevant to behavioral discrimination. The radius was bounded below by one pixel. For images without valid boxes, the image center was used as the fixation point, with a radius equal to 1.5 times half the image diagonal.

For each image pixel, the nearest cluster center was selected by Euclidean distance. The distance to this center and its associated foveal radius were then used to define the spatially varying Gaussian blur intensity as follows:

(3) $$\sigma (p)=\left\{\begin{array}{c} \sigma_{\min}+(\sigma_{r}-\sigma_{\min}){\left(\frac{d(p)}{r(p)+\varepsilon }\right)}^{\gamma_{\mathrm{in}}},d(p)\leq r(p)\\ \sigma_{r}+(\sigma_{\max}-\sigma_{r}){\left(\frac{d(p)-r(p)}{D_{\max}-r(p)+\varepsilon }\right)}^{\gamma_{\mathrm{out}}},d(p)>r(p) \end{array}\right.$$

where *σ_min_* and *σ_max_* represent the minimum and maximum blur intensities, respectively; *σ_r_* denotes the blur intensity at the foveal boundary; *γ_in_* and *γ*_out_ control the blur-growth rates inside and outside the foveal region, respectively; $D_{\max}=\underset{p\in \Omega }{\max}\;d(p)$ represents the maximum distance outside the foveal region; and *ε* is a small constant used to avoid division by zero.

Furthermore, to reduce the influence of background color variation, a saturation attenuation mechanism based on eccentric distance is introduced. The saturation attenuation factor is defined as:

(4) $$a(p)=S_{\min}+(1-S_{\min})\left[1-{\left(\frac{d(p)}{D_{\max}+\varepsilon }\right)}^{\gamma_{s}}\right]$$

where *S_min_* is the minimum saturation-retention ratio, and *γ_s_* is the saturation attenuation coefficient. During augmentation, the original saturation channel *S*(*p*) is updated according to *S*′(*p*) = *S*(*p*) ⋅ *a*(*p*). With this design, target-adjacent regions preserve relatively complete color and structural information, while the color responses of background regions farther from the targets are gradually suppressed, thereby emphasizing juvenile deer bodies and behavior-relevant regions during training.

MBFA was applied offline at the original image resolution using three fixed severity configurations. In the order (σmin, σr, σmax, γin, γout, Smin), the light, medium, and strong settings were (0.3, 2.5, 5.0, 1.2, 0.8, 0.60), (0.6, 4.0, 8.0, 1.5, 1.0, 0.30), and (0.8, 5.0, 10.0, 1.8, 1.2, 0.25), respectively, with Gaussian standard deviations expressed in pixels. All settings used 12 blur levels, a foveal-region enlargement coefficient of 1.5, a saturation exponent of 1.5, and an overlap threshold of 0.3. Parameters within each severity configuration were fixed rather than sampled from continuous distributions.

In the MBFA-only experiment, each training image generated one variant at each severity level. In combined augmentation experiments, one of the three severity levels was selected with equal probability (1/3) for each MBFA variant, and the transformation was applied with probability 1. An augmentation factor of 1 used each training image once; a factor of 1.5 repeated and truncated the training-image list to round (1.5 N) entries before shuffling, where N denotes the original training-image count. Different augmentation types generated separate variants rather than sequential transformations of the same variant. Python 3.11 and NumPy 1.26.4 random generators were initialized with seed 12345 at script startup.

The blur pyramid used 12 standard deviations linearly spaced between σmin and σmax, including both endpoints. Each level was generated using cv2. GaussianBlur with a square kernel of size k = 2 × round(3σ) + 1 and sigmaX = σ; sigmaY and border handling used the OpenCV 4.11.0.86 defaults. The spatial blur map was converted to a fractional pyramid-level index, and adjacent levels were combined by linear interpolation. The interpolated image was clipped to [0, 255] and converted to unsigned 8-bit format before saturation attenuation in HSV space.

The numerical value 10^−6^ was also used in normalization denominators, near-zero checks, and clipping the fractional blur-level index below its upper endpoint. Bounding-box coordinates were clipped to the image boundaries, and boxes with normalized width or height below 10^−6^ were discarded. A separate threshold of σ < 10^−3^ bypassed Gaussian filtering, although this branch was not activated by the three specified severity configurations.

### 2.6. LSARE-Net Architecture

#### 2.6.1. Overall Architecture of LSARE-Net

**Figure 5:**
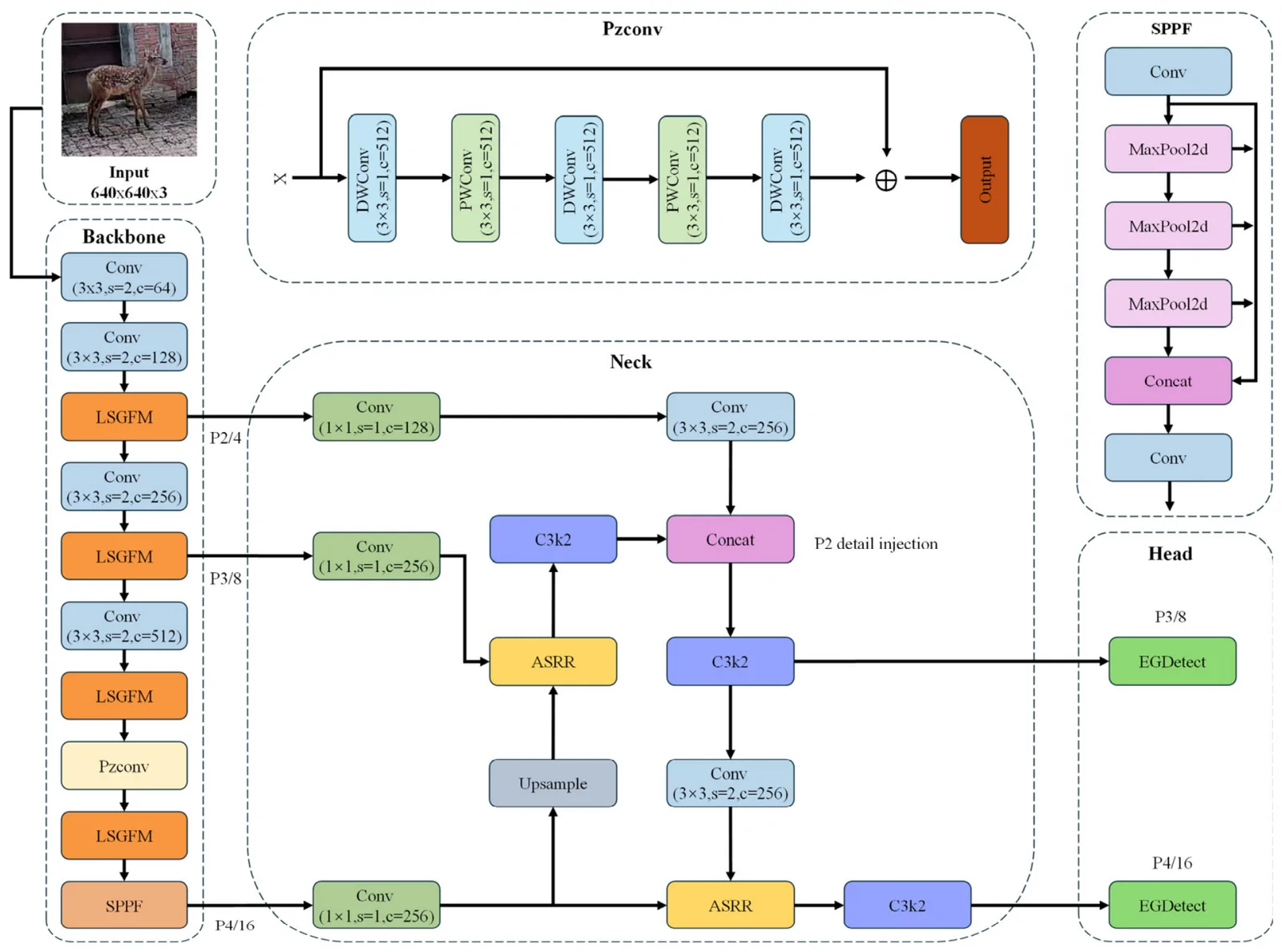
Network architecture of LSARE-Net for juvenile sika deer behavior detection, including the LSGFM backbone, ASRR feature-fusion module and EGDetect head.

Based on the YOLO11 framework, this study constructed a Lightweight Selective Alignment Reorganization and Efficient Detection Network (LSARE-Net) for juvenile sika deer behavior detection. The overall architecture is shown in Figure 5. To address the challenges of small behavioral targets, frequent partial occlusion, and limited resources for edge deployment in real farming environments, LSARE-Net modifies the original YOLO11 architecture in three main components: backbone feature extraction, cross-scale feature fusion, and lightweight detection.

The model used the n-scale configuration, with depth and width multipliers of 0.50 and 0.25, respectively, and a maximum-channel setting of 1024 before width scaling. For a 640 × 640 input, the four LSGFMs produced features of dimensions 32 × 160 × 160, 64 × 80 × 80, 128 × 40 × 40, and 128 × 40 × 40, respectively, expressed as channels × height × width. Both detection inputs contained 64 channels, with spatial resolutions of 80 × 80 and 40 × 40.

In the backbone network, LSARE-Net retains the basic convolutional downsampling structure at the front end of the original YOLO11 to ensure stable extraction of low-level edge, texture, and spatial information. Subsequently, LSGFM is used to replace the main feature extraction units in the original backbone, thereby enhancing the collaborative modeling of local posture cues, texture details, and contextual semantics in juvenile deer. Because LSARE-Net uses a dual-scale P3/P4 detection structure, a Pzconv multi-scale spatial-enhancement unit is introduced at the deep stage of the network to compensate for the reduced high-level contextual receptive field resulting from compression of the P5 branch.

In the feature fusion part, LSARE-Net constructs a bidirectional aligned fusion structure based on ASRR. First, top-down fusion from P4 to P3 is performed to supplement mid-level features with high-level semantic information. Then, shallow P2 details are introduced as an auxiliary branch, allowing edge, contour, and local posture cues to be incorporated into P3 features. Finally, bottom-up feedback fusion from P3 to P4 is conducted to transmit the enhanced local structural information back to the deep branch. For detection output, a dual-scale structure based on P3/8 and P4/16 is adopted, and a lightweight detection head is introduced for prediction, reducing redundant computation caused by low-resolution detection branches. The detailed layer-by-layer architecture and internal module configurations of LSARE-Net are provided in Supplementary File S1.

#### 2.6.2. Lightweight Backbone Based on LSGFM

**Figure 6:**
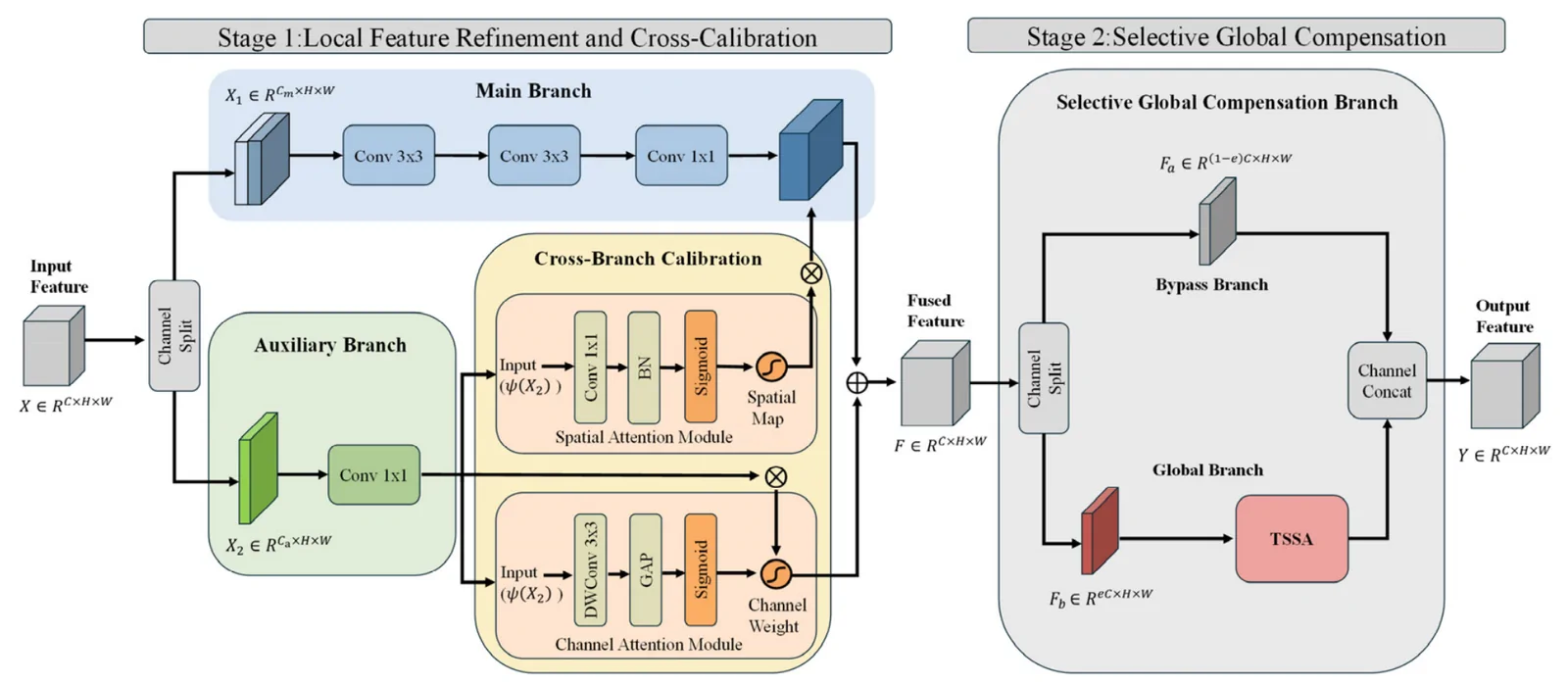
Schematic diagram of the Lightweight Selective Global-aware Fusion Module (LSGFM).

Juvenile deer often occupy small image regions, may be only partially visible, and exhibit substantial posture variation. If the network has insufficient capability to extract edge, texture, and local posture cues, small-target feature responses may become weak, leading to inadequate category discrimination. To address this issue, this study designed a lightweight backbone network based on the Lightweight Selective Global-aware Fusion Module (LSGFM), as shown in Figure 6.

Let the input feature be *X* ∈ *R^C^*^×*H*×*W*^. LSGFM first splits the input feature along the channel dimension into a local branch *X*_1_ and an auxiliary branch *X*_2_. *X*_1_ enhances edge, texture, and posture details through convolutional operations, whereas the auxiliary branch *X*_2_ provides complementary semantic context through channel mapping. In backbone order, the local/auxiliary channel allocations of the four LSGFMs were 24/8, 48/16, 32/96, and 32/96. The local branch applied two 3 × 3 convolutions with stride 1 and padding 1, followed by a 1 × 1 projection, while the auxiliary branch used a single 1 × 1 projection; both branches produced C-channel outputs. Spatial calibration used a C-to-1 pointwise convolution followed by batch normalization and sigmoid, whereas channel calibration used a 3 × 3 depthwise convolution followed by global average pooling and sigmoid. Subsequently, the module constructs a spatial–channel cross-calibration mechanism. The spatial weights generated by the auxiliary branch guide the local branch to focus on the juvenile deer body regions, while the channel weights generated by the local branch enhance the discriminative semantic channels in the auxiliary branch. The cross-calibrated fused feature can be expressed as follows:

*F_f_* = *M_s_*⊗*F_l_* + *M_c_*⊗*F_a_* (5)

where *F_l_* and *F_a_* denote the locally enhanced feature and the auxiliary mapped feature, respectively. *M_s_* and *M_c_* denote the spatial-calibration weight and channel-calibration weight, respectively, and ⊗ represents element-wise multiplication.

After local enhancement and cross-calibration, LSGFM introduces a selective global compensation mechanism. Unlike full-channel global modeling, this mechanism sends only a subset of channels into the lightweight global dependency modeling unit, while the remaining channels are directly preserved, thereby avoiding the additional computational overhead of full-channel attention. Let the fused feature *F_f_* be divided into a retained branch *F*_res_ and a selective global modeling branch *F*_sel_. The final output can be expressed as:

*Y* = *Concat*(*F_res_*,*TSSA*(*F_sel_*)) (6)

Here, the processing branch contained half of the fused channels—16, 32, 64, and 64 channels in the four LSGFMs—and used one TSSA block with a single attention head, while the remaining channels bypassed the block. TSSA used a bias-free input projection, a biased output projection, zero attention and projection dropout, and a token-weight normalization safeguard of 10^−8^; because softmax operated across heads, its output was identically one in this configuration, and feature modulation depended on global channel-wise squared-feature statistics. Residual additions surrounded the attention and feed-forward operations, with the latter comprising 1 × 1 convolutions of dimensions c → 2c → c, batch normalization after both convolutions, and SiLU after the first, followed by concatenation with the bypass branch.

In the lightweight structure adopted in this study, the deep low-resolution detection branch was compressed, and a dual-scale detection output based on P3/P4 was used to reduce redundant computation. However, compression of the deep pathway may weaken the model’s ability to perceive long-range contextual information and high-level spatial structures. To alleviate this issue, a Pzconv multi-scale spatial enhancement unit was introduced at the deep stage of the backbone to expand the effective receptive field of the P4 features. Pzconv sequentially applied 3 × 3, 5 × 5, and 7 × 7 depthwise convolutions, separated by two 1 × 1 pointwise convolutions, while preserving 128 channels throughout. All convolutions used stride 1; the depthwise convolutions included biases and used padding of 1, 2, and 3, respectively, whereas the pointwise convolutions were bias-free and followed by batch normalization and SiLU. The final feature was added to the input through an identity residual connection. This unit enhances the contextual perception capability of P4 deep features without restoring the P5 detection branch, allowing the model to retain effective deep semantic representation under a lightweight architecture.

#### 2.6.3. Aligned Smooth-Weighted Residual Reorganization Fusion Module

**Figure 7:**
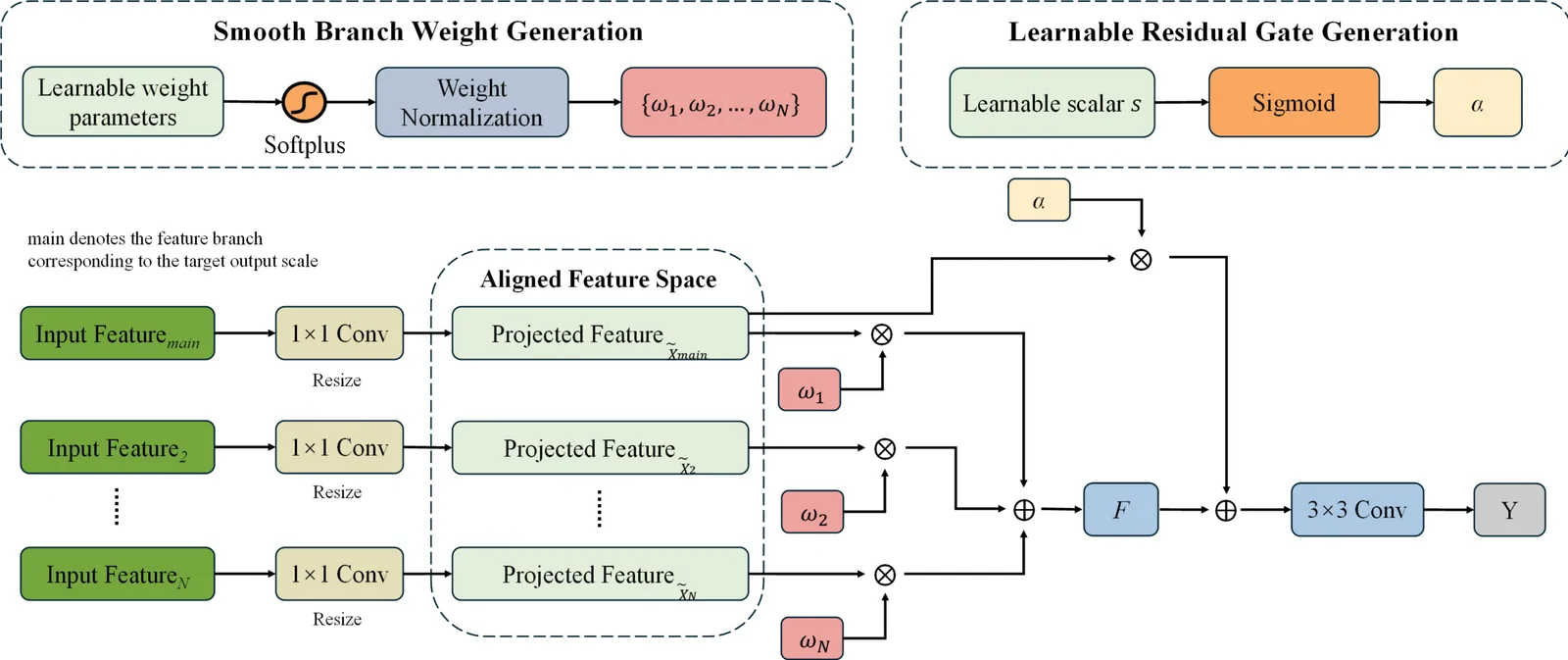
Schematic diagram of the Aligned Smooth-weighted Residual Reorganization Fusion Module (ASRR).

In captive juvenile deer behavior detection, targets are often densely distributed and partially occluded, which can weaken behavior-related local posture cues. Meanwhile, during multi-scale feature fusion, features from different levels usually differ in spatial resolution, channel distribution, and semantic intensity. Existing BiFPN-based methods perform multi-scale feature fusion through non-negative weighting [30]. However, in complex farming scenarios, such methods may still suffer from insufficient cross-level feature alignment, premature suppression of some branch contributions, and dilution of semantic information from the main branch during multi-source fusion. To address these issues, this study proposes an Aligned Smooth-weighted Residual Reorganization Fusion Module (ASRR), whose structure is shown in Figure 7. ASRR reduces feature interference and noise accumulation during multi-source fusion through branch-wise mapping, smooth non-negative weighting, main-branch residual compensation, and local convolutional reorganization. This design improves feature fusion and detection robustness when animals occlude one another and posture cues are partially missing.

Let the multi-branch features input to the current fusion node be denoted as $\{X_{i}{\}}_{i=1}^{N},$ where *N* represents the number of input branches. To reduce the discrepancies in spatial scale and channel dimension among features from different sources, ASRR first performs independent alignment mapping for each branch:

(7) $${\tilde{X}}_{i}=\Phi_{i}\left(X_{i}\right),\;{\tilde{X}}_{i}\in R^{C_{o}\times H\times W}$$

where Φ*_i_* denotes the spatial resizing and channel projection operation for the *i*-th branch.

Subsequently, ASRR introduces branch-level learnable weight parameters *a_i_*. Smooth non-negative weights are generated using the Softplus function and then normalized to obtain the fusion coefficient of each branch:

(8) $$\omega_{i}=\frac{\mathrm{Softplus}(a_{i})}{\sum_{j=1}^{N}\;\mathrm{Softplus}(a_{j})+\varepsilon }$$

Accordingly, the fused feature can be expressed as:

(9) $$F=\sum_{i=1}^{N}\;\omega_{i}{\tilde{X}}_{i}$$

Relying solely on branch-weighted fusion may weaken the core semantics of the main path during multi-source feature mixing. Therefore, ASRR further introduces a main-branch residual compensation mechanism. Let *X_main_* denote the input branch that corresponds to the current output scale and carries the main semantic information and let ${\tilde{X}}_{\mathrm{main}}$ denote its aligned feature. A learnable scalar *s* is introduced, and the residual gating coefficient is generated using the Sigmoid function, i.e., *α* = *σ*(*s*). The final output is expressed as:

(10) $$Y={\mathrm{Conv}}_{3\times 3}\left(F+\alpha {\tilde{X}}_{\mathrm{main}}\right)$$

At both ASRR nodes, each input branch was projected to 64 channels using a 1 × 1 convolution, and a 3 × 3 convolution was applied after weighted fusion and residual addition. Branch-weight parameters were initialized to one and normalized after Softplus activation using ε = 10^−4^. The first input branch served as the residual reference, with its gate parameter initialized to zero, corresponding to an initial sigmoid coefficient of 0.5.

#### 2.6.4. Lightweight EGDetect Head

**Figure 8:**
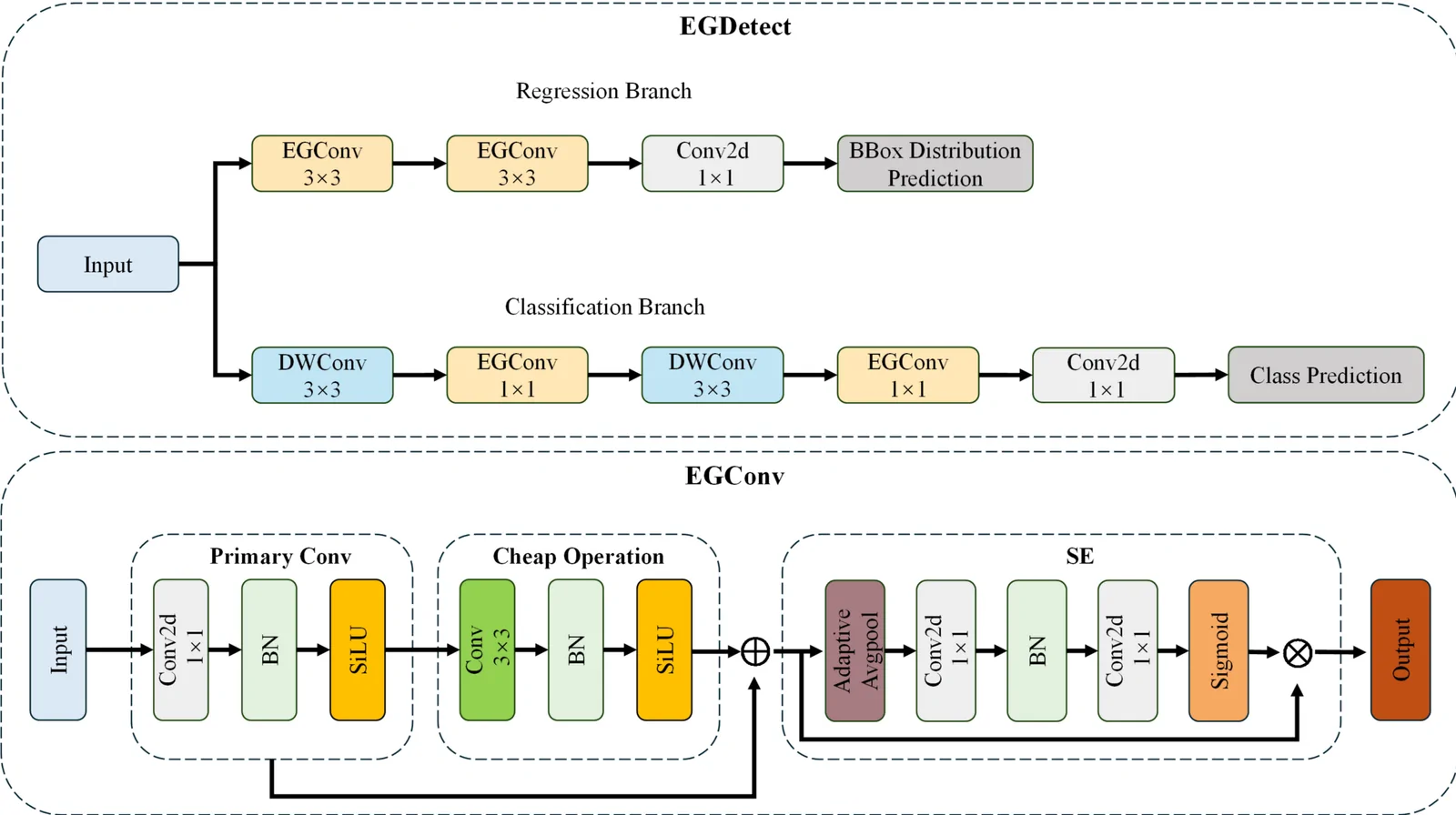
Structure of the EGDetect detection head and EGConv module used for lightweight juvenile sika deer behavior detection.

The detection head is responsible for category prediction and bounding-box regression, and its structural complexity directly affects detection performance and real-time edge inference efficiency. For captive juvenile sika deer monitoring, the detector must handle small targets, occlusion, and posture variation while remaining computationally efficient. Therefore, it is necessary to reduce the computational complexity of the detection head while preserving its decoupled prediction framework. Accordingly, this study introduces a lightweight detection head, EGDetect [31], into the detection network, as shown in Figure 8. Without altering the decoupled prediction framework of the YOLO detection head, several standard convolutional mappings in the detection head are replaced with EGConv. In addition, the SE channel recalibration mechanism and SiLU activation function are incorporated to reduce redundant feature transformations during the prediction stage and enhance channel responses.

EGDetect applies the same regression and classification branch configurations at each input feature scale. The regression branch uses two EGConv blocks with 3 × 3 primary convolutions, followed by a standard 1 × 1 prediction convolution. The classification branch uses two successive pairs of a 3 × 3 depthwise convolution and an EGConv block with a 1 × 1 primary convolution, followed by a standard 1 × 1 prediction convolution. Each EGConv uses a channel-generation ratio of 2, a 3 × 3 depthwise operation, batch normalization, SiLU activation, and SE channel attention with a reduction ratio of 8. The regression and classification branches both used an intermediate width of 64 channels at each detection scale. Each EGConv generated 32 primary and 32 additional channels using bias-free convolutions with stride 1 and same-size padding, while SE used global average pooling and biased 1 × 1 convolutions with dimensions 64 → 8 → 64, followed by SiLU and sigmoid, respectively. No additive residual connection was used within EGConv. The final prediction convolutions output 64 regression channels with reg_max = 16 and five classification channels.

### 2.7. Experimental Settings and Evaluation Metrics

All comparator models and LSARE-Net were trained from scratch using the same dataset split and the common prespecified configuration in Table 4, without model-specific hyperparameter tuning. This design assessed performance under a shared training protocol and did not allocate additional tuning to LSARE-Net. For each model, the best.pt checkpoint selected according to the training framework’s validation fitness criterion was used for test-set evaluation. The common maximum epoch budget and stopping rules standardized the training procedure, but did not guarantee equal computational expenditure or equally favorable convergence across architectures. Model-specific implementation details, preprocessing settings, training epochs, and checkpoint selection are documented in Supplementary File S2.

Model performance was evaluated in terms of detection accuracy, model complexity, and deployment performance. Detection accuracy was assessed using precision, recall, mAP@0.5, mAP@0.5:0.95, and F1-score. Model complexity was evaluated using FLOPs, parameters, and model size, while deployment performance was assessed using FPS and TensorRT engine inference latency. For the object detection task, predicted boxes were matched one-to-one with ground-truth boxes according to the IoU threshold. Successfully matched predictions were regarded as true positives (TP), predictions that failed to match any ground-truth box were regarded as false positives (FP), and ground-truth boxes not matched by any prediction were regarded as false negatives (FN). The main evaluation metrics and their calculation methods are shown in Table 5.

Because this study was an observational computer-vision study based on non-contact surveillance videos, it did not involve experimental treatment allocation, animal-level intervention, or biological response comparison. Therefore, no analysis of variance or treatment-mean comparison was performed. Model performance was evaluated at the annotated target-instance level using the predefined training, validation, and test subsets. All model comparisons were conducted using the same dataset split, training configuration, input size, and evaluation procedure. The results should therefore be interpreted as target-instance-level behavior detection performance under the recorded pen-monitoring conditions, rather than animal-level biological inference or veterinary diagnosis.

## 3. Results

### 3.1. Effect of MBFA Under Complex Imaging Degradation Conditions

Three test conditions—Clean, Color, and Retina—were constructed from the original test images. Clean retained the originals, whereas Color introduced global color and blur perturbations and Retina introduced spatially varying blur and saturation attenuation.

For Color, ColorJitter (brightness/contrast/saturation/hue: 0.08/0.10/0.15/0.04) or RGBShift (per-channel limits: [−18, 18]) was selected with an overall probability of 0.9, followed by HSV shifts (H: 0; S: [−45, −10]; V: [−12, 6]; *p* = 1.0), RandomGamma (gamma_limit = 80–115; *p* = 0.7), and Gaussian or motion blur (5- or 7-pixel kernels; overall *p* = 0.5). Retina used a fixed configuration distinct from all three training-augmentation settings: (σmin, σr, σmax) = (0.35, 2.4, 4.8) pixels, (γin, γout, Smin) = (1.5, 1.0, 0.58), γs = λ = 1.5, τ = 0.3, and 12 blur levels. Ground-truth boxes guided Retina test-image generation but were not provided to the detector during inference.

**Figure 9:**
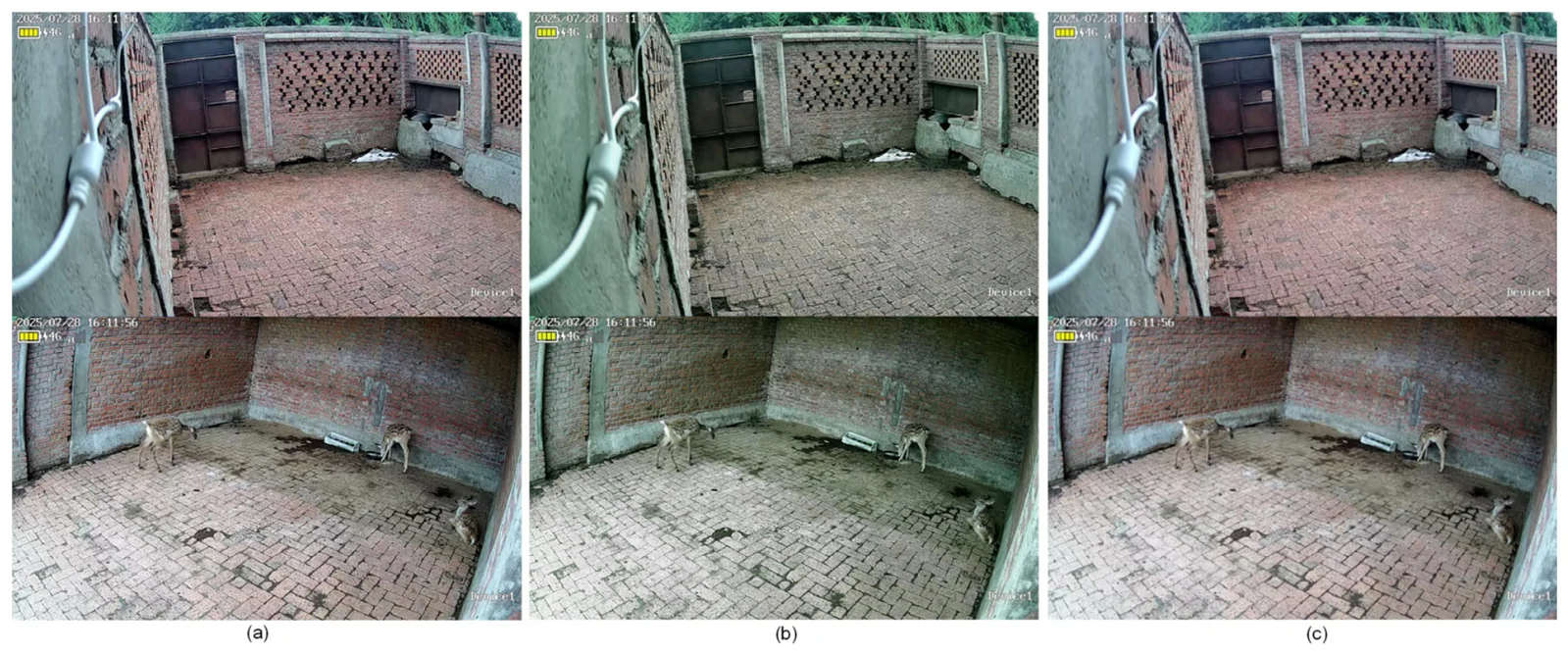
Examples of robustness test scenarios for juvenile sika deer behavior detection: (**a**) Clean; (**b**) Color; (**c**) Retina.

All parameter ranges and application probabilities were specified before inspecting model test results and were not subsequently adjusted based on test performance. Bounding-box coordinates and class labels remained unchanged, and the generated images were used only for robustness evaluation. These conditions assessed robustness to selected MBFA-related synthetic degradations, without establishing general robustness to real-world imaging disturbances. Representative examples are shown in Figure 9.

Based on these test scenarios, YOLO11n was used as the baseline model to compare the effects of conventional appearance augmentation, geometric augmentation, and MBFA under different combinations. All experiments adopted the same dataset split, number of training images, training parameters, and evaluation procedure. mAP@0.5:0.95 was used as the primary evaluation metric.

As shown in Table 6, conventional appearance augmentation mainly improved performance in the Color scenario, whereas geometric augmentation provided a modest improvement in the Retina condition. However, the average gains of these two strategies were limited. After introducing MBFA alone, the mean mAP@0.5:0.95 increased from 77.53% to 78.13%, with the Retina scenario improving from 78.36% to 79.97%. This result suggests that MBFA improves robustness to spatially nonuniform degradation. When MBFA was combined with conventional augmentation strategies, exp7 achieved the highest mean performance, reaching 78.90%. This represented an improvement of 1.37 percentage points over the Baseline and 0.82 percentage points over exp4, which did not include MBFA. Although exp7 was slightly lower than exp3 in the Retina scenario, it performed better in the Clean and Color scenarios as well as in mean performance, suggesting that MBFA is complementary to conventional augmentation. Accordingly, exp7 was used as the training augmentation configuration for the subsequent network-architecture experiments.

**Figure 10:**
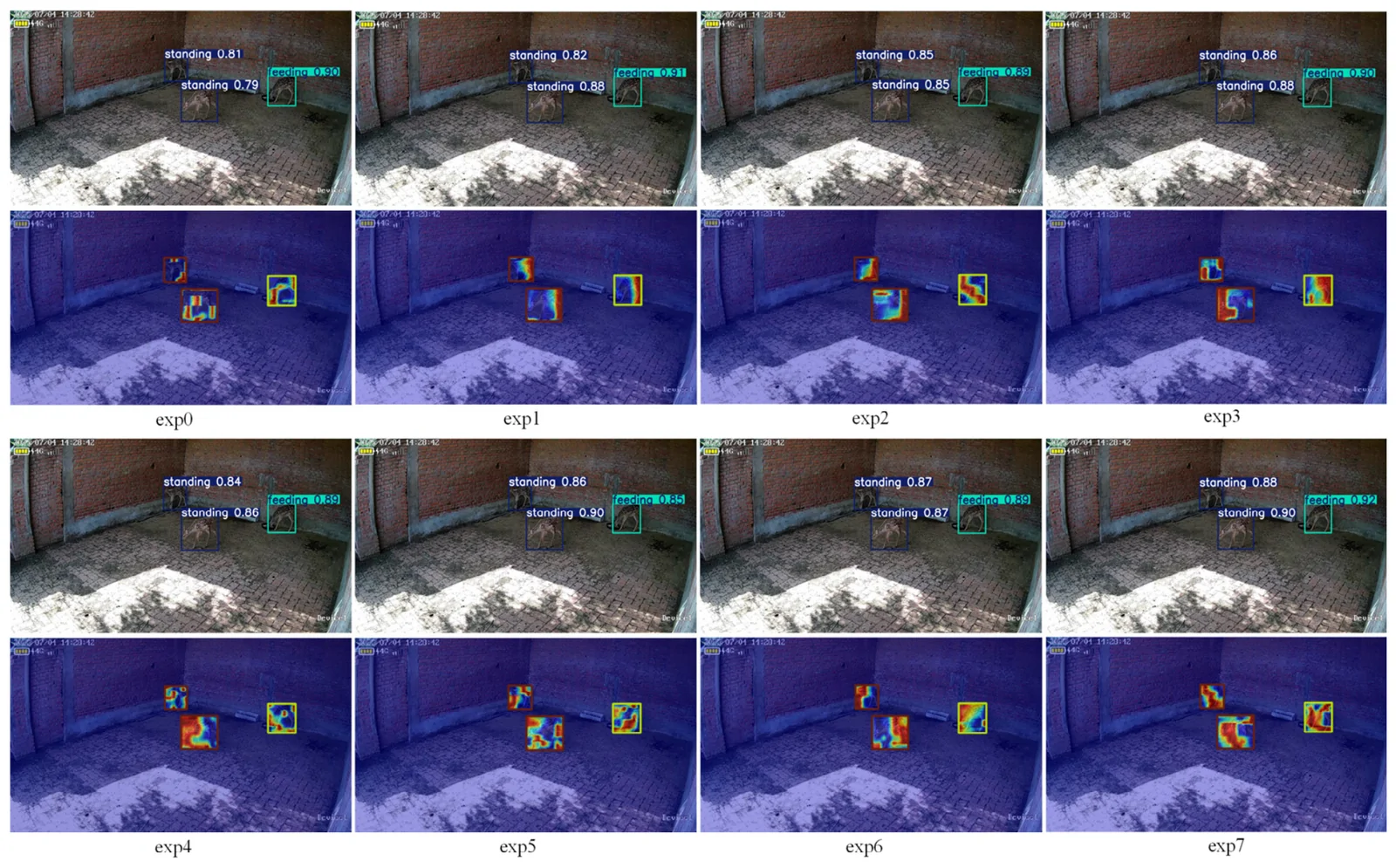
Detection results and Grad-CAM visualization comparison of juvenile sika deer behavior detection under different augmentation strategies.

Furthermore, Grad-CAM was used to visualize model responses under different augmentation strategies [32]. All compared models used the same zero-based target-layer indices (10, 12, 14, 16, and 18). The backpropagation target was the sum of maximum class scores among the highest-ranked 2% of prediction candidates with scores of at least 0.2. Heatmaps were normalized within each predicted bounding box, set to zero outside the boxes, and normalized again after combination; blue-to-red colors indicated increasing relative response rather than absolute attribution strength. Displayed examples were selected according to illumination, target density, occlusion, and proximity to image boundaries. Visualizations from an additional 1170 images were reviewed qualitatively, without predefined assessment criteria or quantitative scoring. Figure 10 provides a qualitative comparison of response patterns within predicted deer regions across augmentation strategies. Because the visualization procedure explicitly suppressed responses outside predicted boxes, these maps do not independently establish reduced background dependence; quantitative performance comparisons are provided in Table 6.

### 3.2. Ablation Analysis of LSARE-Net Modules

To evaluate the contributions of each proposed module to juvenile deer behavior detection performance and model compactness, YOLO11n was used as the Baseline. LSGFM, ASRR, and EGDetect were introduced separately, and different module combinations were further constructed for ablation experiments. All experiments adopted the same dataset split, training parameters, and augmentation configuration.

As shown in Table 7, introducing LSGFM alone increased mAP@0.5 from 92.30% to 93.39% and mAP@0.5:0.95 from 79.02% to 79.58%. Meanwhile, the number of parameters decreased from 2,583,127 to 842,930, and the model size was reduced from 5.2 MB to 1.9 MB. These results suggest that LSGFM reduces parameter redundancy in the backbone while improving local structural and contextual feature representation. After introducing ASRR alone, recall increased to 90.64%, and mAP@0.5:0.95 improved to 79.68%, suggesting that cross-scale aligned fusion helps enhance target detection capability. However, its FLOPs increased to 7.8 G, indicating that feature alignment and reorganization operations introduce additional computational overhead. When EGDetect was used alone, the improvement in mAP was limited, but FLOPs decreased to 5.6 G, indicating that its primary contribution lies in reducing computational redundancy during the prediction stage of the detection head.

From the combined results, the joint use of LSGFM and ASRR increased the model’s mAP@0.5:0.95 to 80.09%, while further reducing the number of parameters to 772,032 and FLOPs to 6.0 G. These results suggest that the lightweight backbone can partially offset the computational overhead introduced by ASRR while complementing cross-scale feature fusion. With EGDetect further incorporated, the full LSARE-Net achieved the highest overall performance in the ablation study, with recall, mAP@0.5 and mAP@0.5:0.95 reaching 91.26%, 93.47%, and 80.24%, respectively. Compared with the Baseline, these metrics were improved by 2.91, 1.17, and 1.22 percentage points, respectively. Meanwhile, FLOPs, the number of parameters, and model size were reduced to 5.4 G, 701,696, and 1.7 MB, corresponding to decreases of 14.29%, 72.84%, and 67.31%, respectively. These results show that LSARE-Net achieves substantial model compression without sacrificing detection accuracy. Instead, it achieves a more favorable balance among detection performance, computational overhead, and model scale.

Although precision decreased slightly relative to the baseline, recall and mAP@0.5:0.95 increased. The higher recall indicates fewer missed detections, which is particularly relevant for screening key behaviors and abnormal cues in continuous monitoring. Event summaries and records can support subsequent manual review of potential false positives.

**Figure 11:**
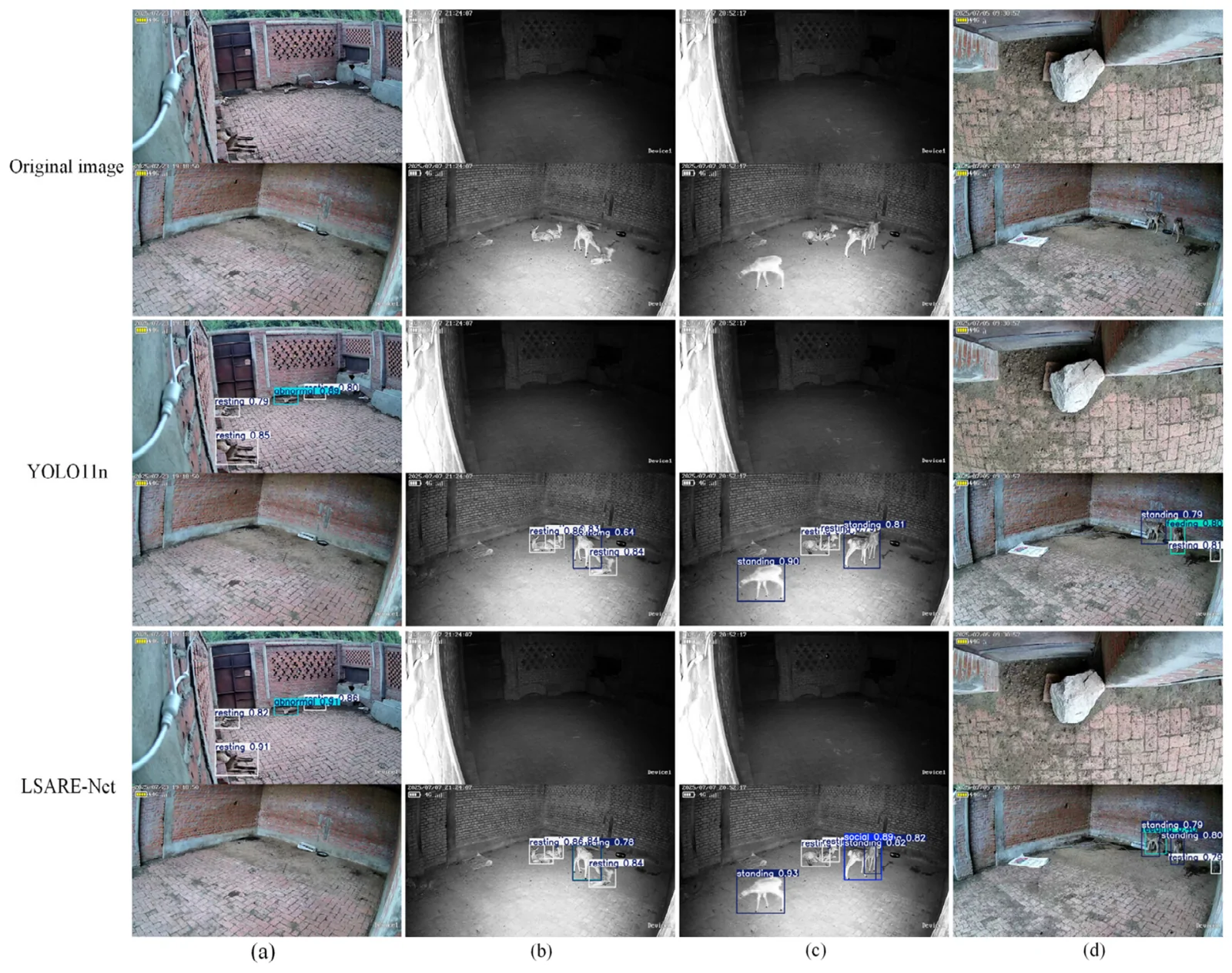
Detection results of LSARE-Net in complex farming scenarios: (**a**) normal illumination scenario; (**b**) low-illumination, long-distance multi-target scenario; (**c**) low-illumination partial-occlusion scenario; and (**d**) partial-occlusion scenario.

**Figure 12:**
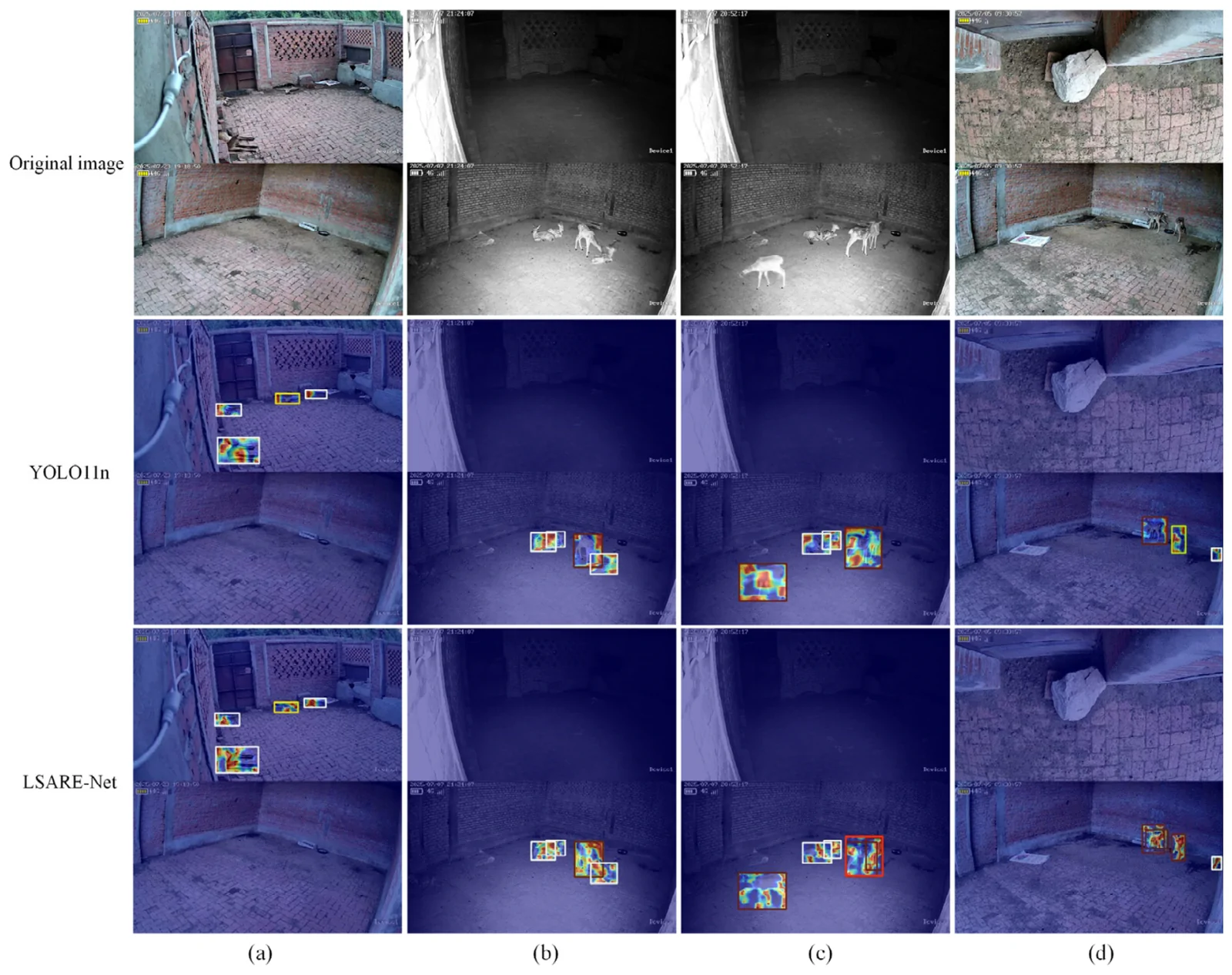
Grad-CAM visualization comparison between YOLO11n and LSARE-Net in complex juvenile sika deer farming scenarios: (**a**) normal illumination scenario; (**b**) low-illumination, long-distance multi-target scenario; (**c**) low-illumination partial-occlusion scenario; and (**d**) partial-occlusion scenario.

Figure 11 and Figure 12 illustrate representative detection outputs and Grad-CAM visualizations of YOLO11n and LSARE-Net under challenging imaging conditions, including low illumination, distant small targets, and partial occlusion. The detection examples illustrate differences in localization and missed detections, while the heatmaps show relative responses within predicted bounding boxes. Because responses outside these boxes were set to zero and normalization was performed separately within each box, the displayed maps cannot establish reduced background dependence or support comparisons of absolute attribution strength between models. Quantitative comparisons of detection performance and computational complexity are reported in Table 7.

### 3.3. Comparison with Representative Detection Models

To evaluate the overall performance of LSARE-Net in juvenile deer behavior detection, this study selected Faster R-CNN [33], SSD [34], RT-DETR-L [35], and YOLO-series models [36,37,38] as comparators. All models were trained and tested under the same dataset split, training augmentation configuration, and experimental environment. FLOPs and parameters were uniformly calculated at an input size of 640 × 640, and Size denotes the corresponding PyTorch 2.2.2 weight file size.

**Figure 13:**
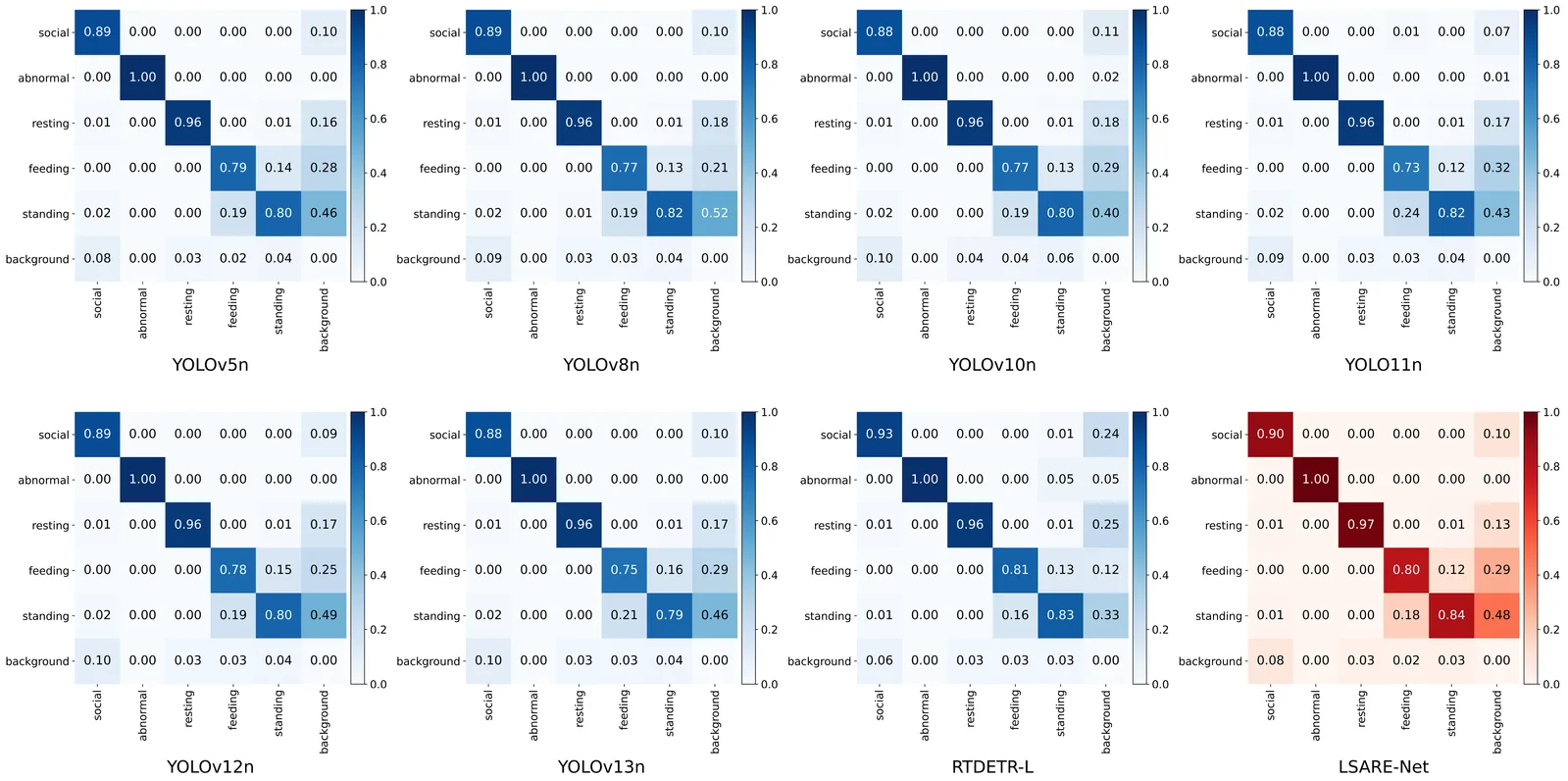
Comparison of confusion matrices of different detection models for juvenile deer behavior detection.

Figure 13 presents the confusion matrices of different models on the test set. Overall, all models performed well on the resting, social, and abnormal categories, whereas greater confusion was observed between feeding and standing. This confusion may reflect similar single-frame appearances during feeding and standing, together with the effects of small target size and partial occlusion. Compared with the other models, LSARE-Net showed a higher concentration of predictions along the diagonal and fewer off-diagonal errors, indicating more consistent class discrimination on the test set.

Detection accuracy and model complexity were compared across models using the shared training configuration (Table 8). Faster R-CNN and SSD achieved mAP@0.5:0.95 values of 58.40% and 22.53%, respectively. Because architecture-specific hyperparameter tuning was not performed, these results characterize performance under the evaluated protocol rather than the best achievable performance of either architecture. Although RT-DETR-L achieved relatively high precision, its mAP@0.5:0.95 was 73.81%, while its FLOPs, parameters, and Size reached 100.6 G, 28.45 M, and 56.4 MB, respectively. This entails substantially greater computational and storage demands, making the model less favorable for continuous edge deployment. By contrast, the lightweight YOLO models achieved a better balance between detection accuracy and model scale. Among them, YOLOv8n and YOLO11n achieved mAP@0.5:0.95 values of 79.10% and 79.02%, respectively, which were higher than those of the conventional detection models and RT-DETR-L, while maintaining relatively low computational overhead. However, the accuracy differences among lightweight YOLO models were limited, and there remained room for further compression in terms of model parameters and size.

Among the evaluated models, LSARE-Net achieved the best overall performance. Its recall, mAP@0.5, mAP@0.5:0.95, and F1-score reached 91.26%, 93.47%, 80.24%, and 90.34%, respectively. Meanwhile, its FLOPs, parameter count, and model size were 5.4 G, 0.70 M, and 1.7 MB, respectively, all lower than those of the other lightweight YOLO models. These results indicate that LSARE-Net does not rely on increasing model scale to improve detection accuracy. Instead, through the coordinated design of lightweight backbone feature enhancement, cross-scale aligned fusion, and a lightweight detection head, LSARE-Net achieves a more favorable balance among detection performance, computational overhead, and model size.

Class-specific results are summarized in Table 9. Precision, recall, and TP/FP/FN counts were calculated at a confidence threshold of 0.25 using same-class, one-to-one matching at IoU ≥ 0.50, whereas AP was calculated across confidence thresholds. For abnormal recumbency, the model produced 212 true positives, one false positive, and no false negatives on the sampled test images.

### 3.4. Performance Variability Across Independent Training Runs

To assess performance variability across random initializations, YOLO11n and LSARE-Net were each trained from scratch using six random seeds (0–5), while keeping the dataset split, training configuration, and data augmentation strategy unchanged. For each run, the checkpoint selected according to validation fitness was evaluated on the same test set. The individual test results and model complexity are reported in Table 10.

Across the six runs, YOLO11n achieved mean ± sample standard deviation values of 90.84 ± 0.89%, 88.90 ± 0.79%, 92.38 ± 0.23%, 78.67 ± 0.35%, and 89.82 ± 0.22% for precision, recall, mAP@0.5, mAP@0.5:0.95, and F1, respectively. The corresponding values for LSARE-Net were 91.16 ± 0.83%, 89.71 ± 1.15%, 93.58 ± 0.32%, 79.99 ± 0.36%, and 90.40 ± 0.45%.

To assess whether the performance differences between LSARE-Net and YOLO11n were consistent across training runs, results were paired by seed identifier under the same dataset split and training protocol. For each metric, LSARE-Net minus YOLO11n was calculated within each seed, yielding six paired differences as the units of analysis. Two-sided paired *t*-tests were applied under the assumptions of independence across seed pairs and approximate normality of the differences. Individual, unadjusted 95% confidence intervals for the mean paired differences were calculated using the t distribution with five degrees of freedom, and *p*-values were adjusted across the five metrics using the Holm procedure.

LSARE-Net achieved mean paired improvements of 1.203 percentage points in mAP@0.5 (95% CI: 0.866–1.541) and 1.320 percentage points in mAP@0.5:0.95 (95% CI: 1.138–1.502). Both improvements remained statistically significant after Holm correction, with adjusted *p*-values of 0.001037 and 0.000041, respectively. The mean paired differences in precision, recall, and F1 were 0.315 percentage points (95% CI: −1.191–1.821), 0.812 percentage points (95% CI: −1.032–2.656), and 0.582 percentage points (95% CI: 0.003–1.161), respectively. These three differences were not statistically significant after correction (adjusted *p* = 0.6184, 0.6184, and 0.1480, respectively).

The higher mAP@0.5 and mAP@0.5:0.95 values achieved by LSARE-Net for all six evaluated seeds, together with the paired analysis, support the repeatability of the observed mAP advantage over YOLO11n under the studied conditions.

### 3.5. Within-Scene Model Comparison and Visualization Analysis in an Additional Pen

**Figure 14:**
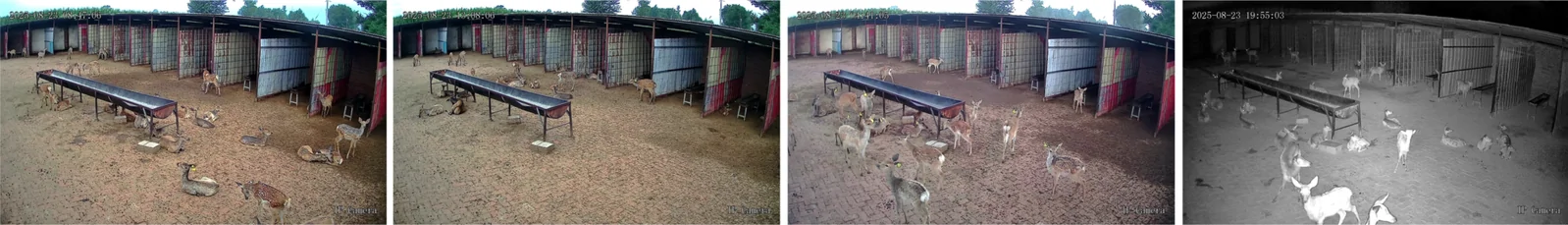
Representative samples from the additional-scene dataset, including low illumination, dense multi-target distribution, partial occlusion and viewpoint variation.

An additional dataset was collected from a different pen at the same deer farm between 1 and 10 September 2025. The 1400 original images were randomly partitioned at the image level into 973 training images, 283 validation images, and 144 test images, without grouping by source video or recording date. No data augmentation was applied to this additional dataset. A single annotator completed the annotations and self-review for resting, feeding, and standing using the criteria in Table 2. Both YOLO11n and LSARE-Net were trained from scratch on the training subset from the additional pen and evaluated on the test subset from that same pen, without loading pretrained or main-dataset weights. This experiment therefore compared the two architectures within the additional scene and did not assess transfer of the main-dataset-trained models. The overall and class-specific detection performance and model complexity of YOLO11n and LSARE-Net after training within the additional scene are summarized in Table 11. Representative examples showing variations in illumination, background, target distribution, and viewpoint are presented in Figure 14.

**Figure 15:**
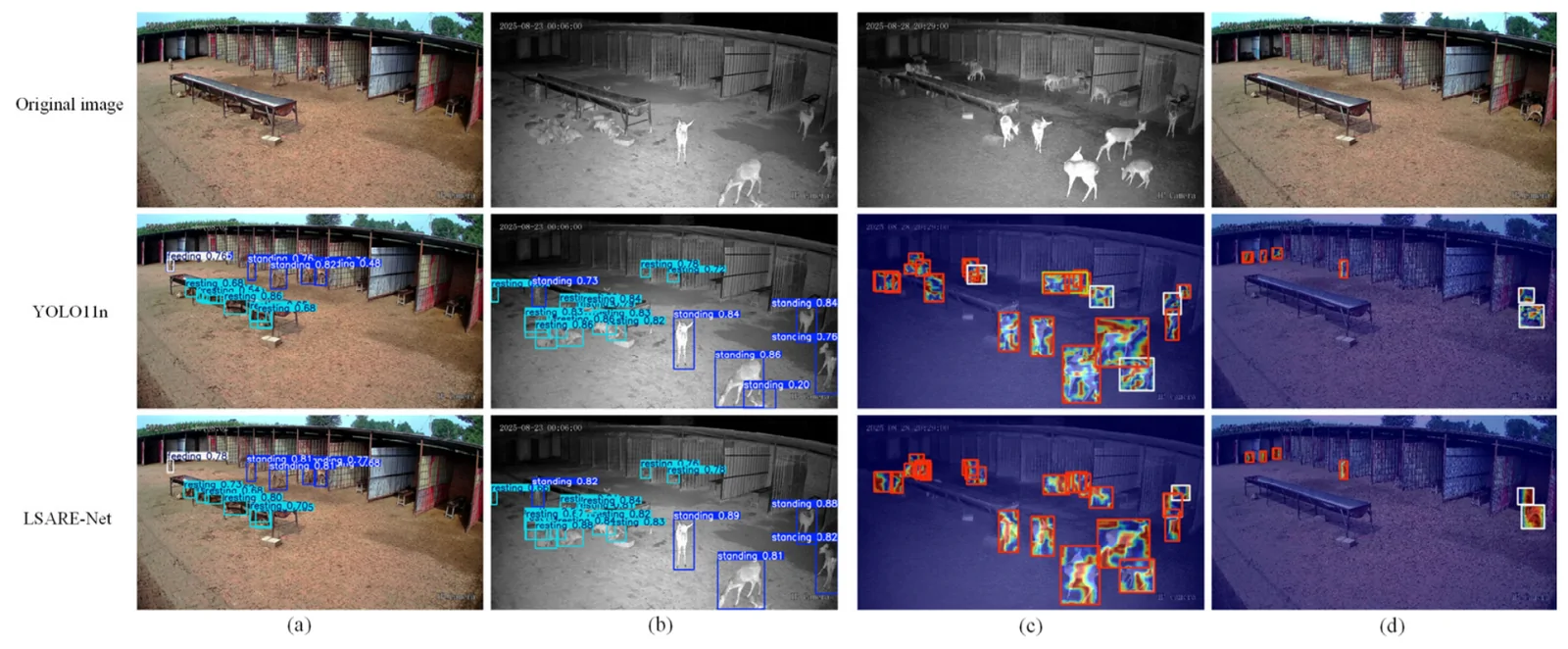
Detection outputs and Grad-CAM visualizations of YOLO11n and LSARE-Net after training within the additional scene: (**a**) normal-illumination scene; (**b**) low-illumination scene; (**c**) low-illumination scene with densely distributed targets; and (**d**) scene with sparsely distributed targets near image boundaries.

Figure 15 compares representative detection outputs and Grad-CAM visualizations of YOLO11n and LSARE-Net after training within the additional scene. In the selected examples involving low illumination, dense target distribution, and partial occlusion, LSARE-Net showed fewer visible detection errors for some occluded targets and targets near image boundaries. The heatmaps illustrate relative response patterns within predicted bounding boxes using the masking and normalization procedure described in Section 3.1. Because responses outside these boxes were explicitly set to zero, the visualizations do not establish reduced background dependence or support comparisons of absolute attribution strength between models.

### 3.6. Edge Deployment Performance on Jetson AGX Orin

To evaluate the deployment feasibility of LSARE-Net in real-time edge-monitoring scenarios, the trained model was deployed on the NVIDIA Jetson AGX Orin platform. During deployment, the PyTorch weights were first exported to the ONNX format and then converted into a TensorRT engine file to reduce inference latency and improve edge-device execution efficiency. The deployment environment is shown in Table 12.

To evaluate the inference performance of different models on the edge device, all models were tested using the same input size and inference workflow. Detection accuracy in Table 13 refers to the test results obtained after TensorRT engine inference.

As shown in Table 13, LSARE-Net maintained favorable detection performance after edge deployment, achieving mAP@0.5 and mAP@0.5:0.95 values of 93.20% and 79.61%, respectively, both of which were higher than those of the original YOLO11n and other lightweight YOLO models. Meanwhile, the FLOPs and number of parameters of LSARE-Net were 5.4 G and 0.70 M, respectively, and the TensorRT engine file size was 5.7 MB. These results indicate that LSARE-Net provides a favorable resource profile in terms of computational cost, parameter count, and deployment file size.

In terms of inference speed, YOLOv10n achieved the highest FPS, but its detection accuracy was lower than that of LSARE-Net. The average inference speed of LSARE-Net was 62.64 FPS. Although this value was lower than that of some lightweight YOLO models, it remained sufficient for real-time video processing. It should be noted that edge-device FPS is affected not only by FLOPs but also by operator type, memory-access overhead, and the degree of TensorRT optimization. In LSARE-Net, LSGFM and ASRR improve local–global feature modeling and cross-scale aligned fusion, but they also introduce additional operator scheduling and feature-map reorganization overhead. Therefore, the FPS does not increase linearly with the reduction in FLOPs. Considering detection accuracy, model size, and real-time performance, LSARE-Net achieves a favorable accuracy–efficiency balance for edge-based juvenile deer behavior recognition.

Inference latency was measured on the NVIDIA Jetson AGX Orin using TensorRT FP16 engines with an input size of 640 × 640 and a batch size of 1. The device operated in MAXN mode, with CPU and GPU clocks locked at the maximum frequencies permitted by this configuration. Each model underwent 200 warmup iterations, excluded from timing, followed by 500 timed inference iterations in a single benchmark run. Timing covered TensorRT engine execution only, excluding data loading, preprocessing, postprocessing, and host–device data transfers. Mean latency was selected as the primary summary statistic, with the median, P90, P95, and P99 additionally reported to characterize the latency distribution. The TensorRT engine inference latency results for all compared models are summarized in Table 14.

**Figure 16:**
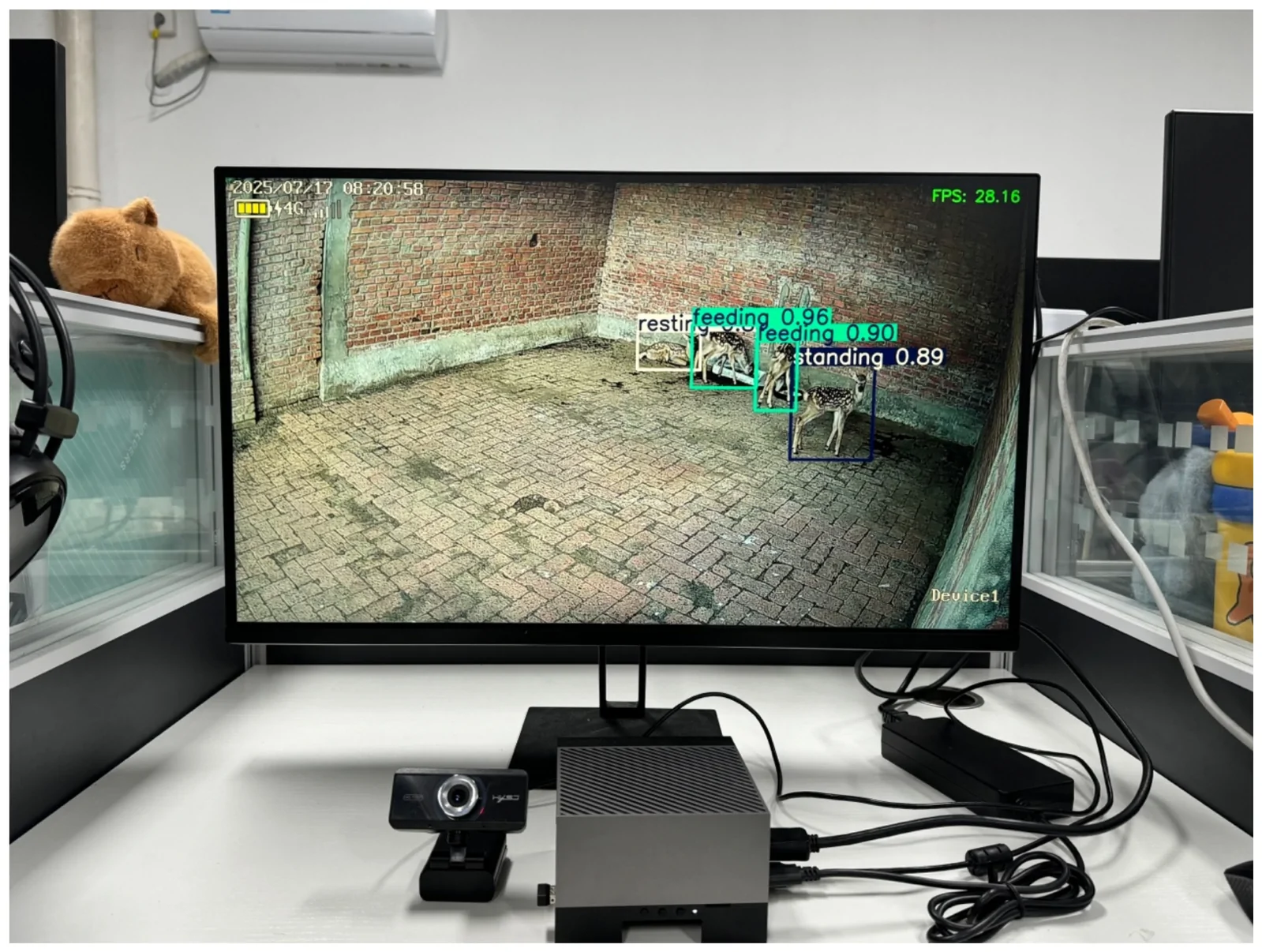
Real-time edge deployment of LSARE-Net for juvenile sika deer behavior recognition on the NVIDIA Jetson AGX Orin platform.

To demonstrate the practical deployment performance, a real-time edge-monitoring interface based on LSARE-Net was further constructed. As shown in Figure 16, the system can detect juvenile deer targets in input videos in real time and display the predicted behavioral category, confidence score, and current processing frame rate.

The FPS shown in the figure is the frame rate displayed during operation of the complete visualization system. In addition to model inference, it includes extra overhead from video reading, result rendering, and interface display. Therefore, it differs from the model inference FPS obtained under the standardized benchmark protocol in Table 13.

### 3.7. Monitoring-System Output for Behavioral Data Acquisition

**Figure 17:**
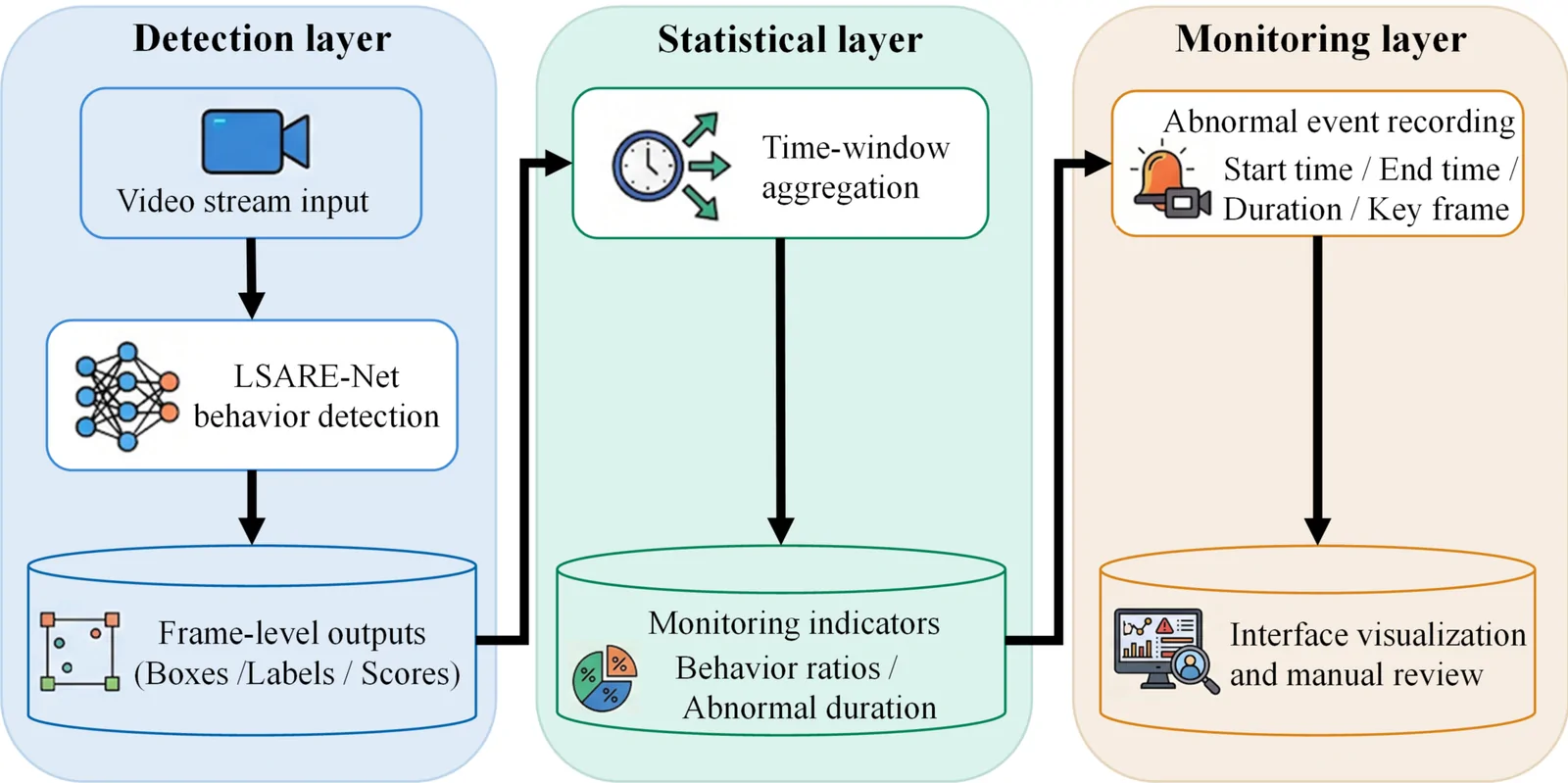
Workflow for converting frame-level juvenile sika deer behavior detections into structured behavioral monitoring variables.

To support continuous behavioral monitoring, this study developed a workflow that converts frame-level LSARE-Net outputs into structured monitoring variables, as shown in Figure 17. Bounding boxes, behavioral categories, and confidence scores were processed within sliding time windows to generate behavioral proportions, abnormal-presence duration, and scene-level abnormal-recumbency event records.

Video frames were processed sequentially with an inference image size of 640, a confidence threshold of 0.25, and an NMS IoU threshold of 0.70. Time was derived from the frame index and nominal video frame rate, with 25 frames/s used when the reported value was nonpositive. Behavioral summaries used a rolling window of int(60f) frames and were updated every max(1, int(f)) frames, where f denotes the video frame rate. During startup, all available frames were used. For each category, the behavioral proportion was calculated as its accumulated detection count divided by the total detection count within the window; all proportions were set to zero when no detections were present. Missing detections were not interpolated or replaced. These values therefore represent detection-instance proportions rather than individual behavioral time budgets.

Abnormal-presence duration within each window was calculated as the number of frames containing at least one abnormal-recumbency detection divided by f. A qualifying frame initiated a scene-level event, which remained active until more than int(3f) consecutive frames contained no abnormal detection. Concurrent abnormal detections were pooled at the scene level without individual tracking or an additional spatial-overlap criterion. Event duration was defined by the interval between the first and last abnormal-positive frames, excluding the trailing absence interval. Each completed record stored the start and end frames and times, duration, maximum abnormal-detection confidence, and an annotated image captured at event onset. Active events were finalized when video processing ended.

**Figure 18:**
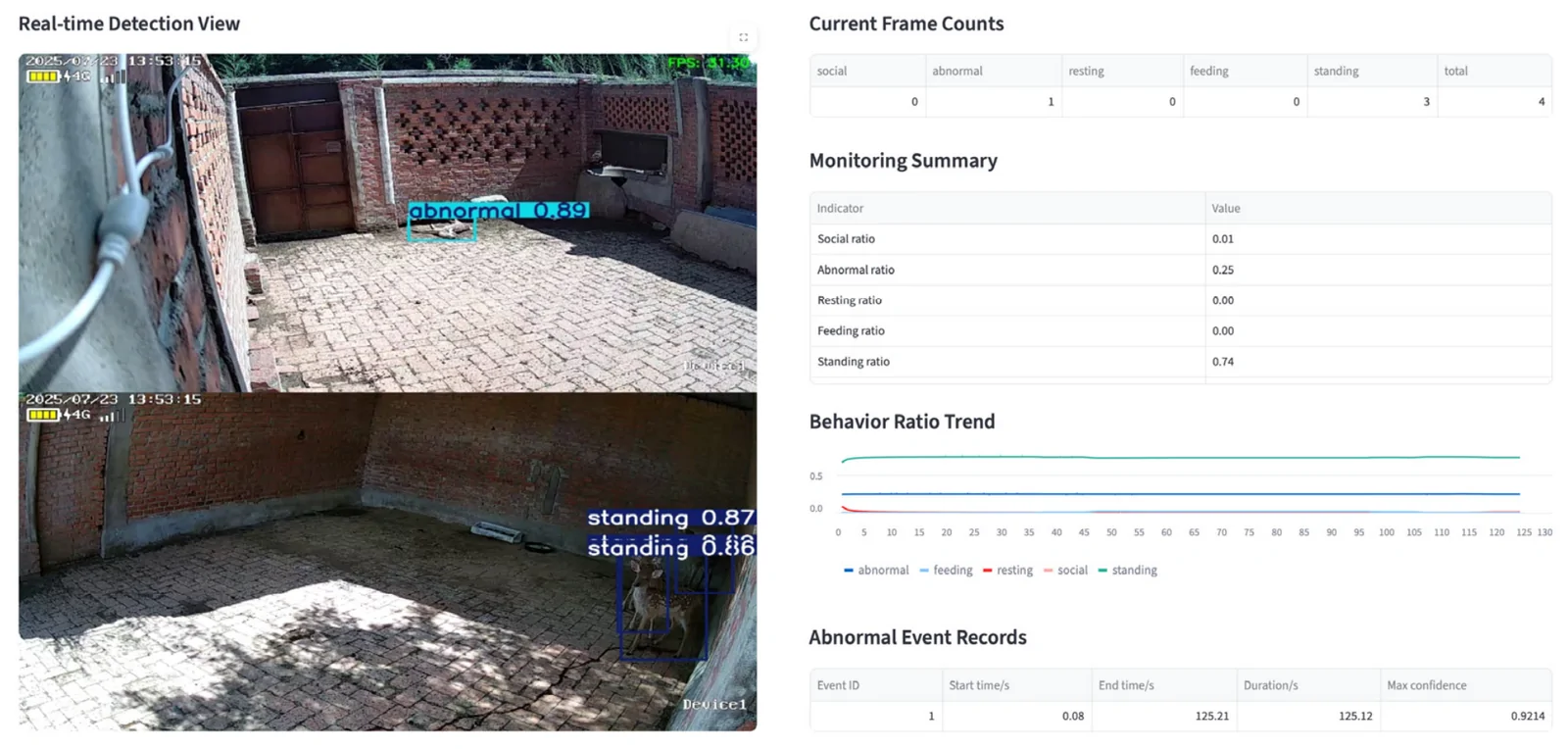
Interface of the juvenile deer behavior recognition and abnormal-state auxiliary monitoring system. The colors of the detection boxes on the left are used to distinguish detected targets, while the colors on the right indicate different behavior categories.

An auxiliary monitoring interface for juvenile deer behavior recognition and abnormal-state review was further developed based on this workflow, as shown in Figure 18. The interface includes real-time detection, current-frame behavior counts, monitoring summaries, behavioral-proportion trends, and abnormal-event records. It presents the generated detection and event information in a traceable and reviewable format for abnormal-cue screening and farm-management review.

A qualitative review covered six 30-min videos, totaling 3 h, and included one observed abnormal-recumbency episode lasting approximately 2 min. Because the evaluator examined the system outputs while checking the source videos, this review was not an independent event-annotation assessment, and behavioral-proportion, duration, and event-detection errors were not independently quantified. The event-triggering parameters and event records are part of the operational prototype; event-level sensitivity, precision, false-alarm rate, and duration accuracy remain to be validated.

## 4. Discussion

Continuous behavioral monitoring of captive juvenile sika deer is challenging because abnormal cues are often subtle, intermittent and difficult to capture through discontinuous manual inspection. In this context, the main value of the proposed workflow lies not only in detecting juvenile deer in individual frames but also in converting frame-level outputs into window-based behavioral proportions, abnormal-presence duration and reviewable event records. The frame-level detection results and integrated monitoring prototype demonstrate the technical feasibility of automated juvenile deer behavior analysis under the studied pen conditions. With the main dataset limited to six deer in one pen, broader generalizability, event-level reliability, and potential benefits for health management and animal welfare require further evaluation.

Previous studies have explored motion-focused global–local modeling for cattle behavior recognition [19], YOLOX combined with SCTS-SlowFast for group-housed pig behavior detection [20], and Sheep-YOLO for lightweight recognition of lamb behavior and vitality states [15]. In the present fixed-camera juvenile sika deer monitoring task, distant individuals occupy small image regions. Inter-animal occlusion further obscures posture cues. These conditions require preservation of local visual detail while meeting the computational constraints of edge deployment. To address these challenges, the proposed framework combines target-guided augmentation, local feature enhancement, cross-scale fusion, and a compact detection head, with deployment evaluated on the NVIDIA Jetson AGX Orin platform.

The results showed that LSARE-Net improved detection performance while maintaining a compact model structure. Compared with YOLO11n, LSARE-Net increased recall and mAP@0.5:0.95 while reducing the number of parameters and model size, indicating that the improvement was not achieved by simply expanding model capacity. Instead, lightweight feature extraction, cross-scale aligned fusion and lightweight detection output jointly improved the representation of behavior-related cues in scenes containing small targets, partial occlusion, and background clutter. For abnormal cue screening in continuous video monitoring, improved recall is particularly important because missed detections may lead to incomplete event records. Time-window summaries and abnormal-event records can support subsequent manual review of potential false detections.

The main inter-class confusion occurred between feeding and standing: LSARE-Net reduced feeding-to-standing errors from 0.24 to 0.18 compared with YOLO11n, while standing-to-feeding confusion remained at 0.12. This remaining confusion may reflect similar single-frame appearances during head lowering, ground sniffing, and feeding pauses, motivating further evaluation of temporal information. Resting and abnormal recumbency showed little confusion in the sampled test set. The abnormal-class AP and confusion-matrix results describe performance on sampled test frames that may be correlated. Without cluster-adjusted uncertainty estimates, these results alone do not establish reliability across independent abnormal-recumbency events. In practical monitoring, false negatives could leave abnormal-recumbency cues unrecorded and delay manual assessment, whereas false positives could increase unnecessary review and on-site checks.

In practical deployment, occlusion, crowding, and day–night illumination changes can obscure behavioral cues and cause missed or inconsistent detections. These errors can affect behavioral proportions and event-duration estimates, and temporal aggregation alone may not eliminate persistent false detections. Monitoring outputs therefore require validation against manually reviewed continuous videos under varied camera placements and husbandry conditions.

MBFA was designed to preserve target-adjacent information while degrading more distant background regions, whereas LSGFM combines local structural cues with contextual information to support behavioral discrimination. ASRR aligns and fuses features across scales for small and occluded targets, whereas EGDetect reduces prediction-head complexity through efficient feature generation and SE channel reweighting. The combined configuration improved mAP@0.5:0.95 from 79.02% to 80.24% while reducing parameters from 2.58 M to 0.70 M, supporting a more favorable accuracy–size trade-off without independently proving each proposed mechanism.

The image-level random split did not group images by source video or recording date, so frames from the same or related recordings may occur across subsets. The results support model comparison under the evaluated conditions, but do not by themselves establish generalization to independent recordings or temporally separated data.

All labels were produced and self-reviewed by one annotator without an independent reliability assessment. Class-specific results, particularly the near-perfect performance for abnormal recumbency, should be interpreted with this limitation in mind. The abnormal category in this study was restricted to visually observable abnormal recumbency and did not cover other types of abnormal behavior. Accordingly, the reported performance for this category applies only to abnormal-recumbency detection and should not be interpreted as evidence of general abnormal-behavior recognition or veterinary diagnostic capability.

## 5. Conclusions

This study proposed a lightweight machine-vision system for real-time behavioral monitoring of captive juvenile sika deer under complex enclosure conditions. By combining MBFA-based training augmentation, LSARE-Net detection, and an edge-based monitoring workflow, the proposed framework improved target-instance-level behavior recognition while maintaining low model complexity. Under the main dataset’s image-level random split, LSARE-Net achieved 91.26% recall, 93.47% mAP@0.5 and 80.24% mAP@0.5:0.95, with 0.70 M parameters and a model size of 1.7 MB. After TensorRT conversion, the model reached 62.64 FPS on the NVIDIA Jetson AGX Orin platform, supporting real-time edge deployment. The system further converted frame-level detections into behavioral proportions, abnormal-presence duration and reviewable event records. These findings indicate that the proposed system can support continuous screening of behavioral cues and manual review in captive juvenile deer management, but they should not be interpreted as evidence for animal-level biological inference or veterinary diagnosis.

Future work will expand data collection across pens, seasons, and geographic regions and evaluate performance on animals and sites excluded from training. Future dataset partitions will assign each complete recording session to a single subset and introduce temporal separation between subsets to better assess transfer to independent recordings. Monitoring outputs will also be evaluated against continuous videos annotated without access to system predictions, including multiple distinct abnormal-recumbency episodes. This will extend the present frame-level evaluation toward event-level validation. Using predefined temporal matching criteria, the evaluation will quantify errors in behavioral proportions and event durations, event-level precision and recall, and false alarms per hour.

## Figures and Tables

**Table 1:** Comparison of publicly available deer-related data resources and their limitations for captive juvenile sika deer behavior detection.

Dataset	Task	Annotation	Behavior Labels	Limitation for This Study
Deer Cameratraps	Detection	Bounding box	No	Single deer target detection; no behavior-state labels
Deer Dataset	Detection	Bounding box	No	Single deer class only; no behavior-state labels
Missouri Camera Traps	Classification/Detection	Image-level labels and limited boxes	No	Multi-species camera-trap data; not designed for captive behavior detection

**Table 2:** Criteria for behavioral classification and annotation of five juvenile sika deer behaviors.

Category	Classification and Annotation Criteria	Potentially Confused Category
resting	The deer is recumbent with its limbs naturally folded beneath or alongside the body and a relaxed posture, generally maintaining a supported head and neck position. Low activity or head lowering alone is not sufficient for classification as abnormal.	abnormal
standing	The juvenile deer stands on all four limbs without clear evidence of feeding, active social interaction, or abnormal behavior. This includes ground sniffing without feed intake and standing near another deer without clear interaction, whereas head-raised chewing during a feeding bout is classified as feeding.	feeding/social
feeding	The deer shows observable feed intake or chewing associated with feeding, including brief head-raised periods when chewing continues. A lowered head or proximity to the feeding trough or ground alone is insufficient for classification as feeding; ground sniffing without evidence of feed intake is classified as standing.	standing
social	The deer engages in a clearly observable interaction with another deer, such as directed physical contact, head-butting, or an approach accompanied by an observable interactive response. Spatial proximity alone, without clear interaction, is not classified as social.	standing
abnormal	Abnormal recumbency is identified by the combined presence of splayed limbs, a lowered head and neck without the supported posture typical of normal resting, and an atypical overall body posture.	resting

**Table 3:** Class-specific image and target-instance counts across dataset subsets before and after training augmentation.

Dataset	Count Type	Resting	Standing	Feeding	Social	Abnormal	Total
Training (before augmentation)	Images	963	933	787	529	340	2063
Training (before augmentation)	Instances	1927	1571	974	535	340	5347
Training (after augmentation, including originals)	Images	3852	3732	3148	2116	1360	8252
Training (after augmentation, including originals)	Instances	7708	6284	3896	2140	1360	21,388
Validation	Images	1101	1046	947	561	415	2358
Validation	Instances	2112	1821	1226	562	415	6136
Test	Images	526	514	430	322	212	1179
Test	Instances	1008	912	539	325	212	2996

**Table 4:** Training parameter settings for juvenile sika deer behavior detection models.

Parameter	Value
Image size	640 × 640
Maximum training epochs	300
Batch size	16
Optimizer	SGD
Initial learning rate	0.01
Final learning-rate factor	0.01
Momentum	0.937
Weight decay	0.0005
Automatic mixed precision	True
Warm-up epochs	3
Warm-up momentum	0.8
Warm-up bias learning rate	0.1
Early stopping patience	100 epochs
Deterministic mode	True

**Table 5:** Evaluation metrics and calculation methods for model performance assessment.

Evaluation Indicator	Full Name	Computational Equation
*P*	Precision	$P=\frac{\mathrm{TP}}{\mathrm{TP}+\mathrm{FP}}\times 100\%$
*R*	Recall	$R=\frac{\mathrm{TP}}{\mathrm{TP}+\mathrm{FN}}\times 100\%$
*AP*	Average Precision	$\mathrm{AP}=\int_{0}^{1}\;P(r)\mathrm{dr}$
*mAP*	Mean Average Precision	$\mathrm{mAP}=\frac{1}{n}\sum_{i=1}^{n}\;AP_{i}$
*F*_1_	F1-score	$F1_{c}=\frac{2P_{c}R_{c}}{P_{c}+R_{c}},F1_{\mathrm{macro}}=\frac{1}{K}\sum_{c=1}^{K}\;F1_{c}$
*FPS*	Frames Per Second	$\mathrm{FPS}=\frac{1000N}{T_{\mathrm{total}}}$
*Size*(*MB*)	Model Size	$\frac{\mathrm{Parameters}\times 4}{1024^{2}}$

Note: *P*(*r*) denotes precision at recall *r*. mAP@0.5 represents mean AP at an IoU threshold of 0.5, whereas mAP@0.5:0.95 averages AP over IoU thresholds from 0.5 to 0.95 in steps of 0.05. In the F1 equation, *K* denotes the number of evaluated categories, and overall *F*_1_ is the unweighted mean of class-specific *F*_1_ scores rather than the harmonic mean of overall precision and recall; class-specific *F*_1_ is defined as zero when both precision and recall are zero. In the FPS equation, *N* denotes the total number of processed frames and *T*_total_ denotes their total measured processing time in milliseconds. Size denotes model-file storage size, while TensorRT engine size, FPS, and engine inference latency are reported separately in the deployment experiments. FLOPs and Parameters were calculated using the same input size in the Ultralytics framework, with FLOPs expressed in billions of operations (G).

**Table 6:** Comparison of different augmentation strategies for juvenile sika deer behavior detection under Clean, Color and Retina test scenarios.

Experimental Group	Augmentation Strategy	Clean (%)	Color (%)	Retina (%)	Mean (%)
exp0	Baseline	77.66	76.57	78.36	77.53
exp1	Baseline + appearance	77.44	77.74	78.17	77.78
exp2	Baseline + geometric	77.29	76.88	78.93	77.70
exp3	Baseline + MBFA	77.61	76.80	79.97	78.13
exp4	Baseline + appearance + geometric	78.28	77.73	78.23	78.08
exp5	Baseline + appearance + MBFA	78.50	77.32	78.55	78.12
exp6	Baseline + geometric + MBFA	78.58	77.34	79.42	78.45
exp7	Baseline + appearance + geometric + MBFA	79.02	77.98	79.69	78.90

**Table 7:** Ablation comparison of LSARE-Net modules for juvenile sika deer behavior detection.

LSGFM	ASRR	EGDetect	P (%)	R (%)	mAP@0.5 (%)	mAP@0.5:0.95 (%)	F_1_ (%)	FLOPs (G)	Parameters	Size (MB)
			91.84	88.35	92.30	79.02	90.04	6.3	2,583,127	5.2
√			89.11	90.01	93.39	79.58	89.55	6.2	842,930	1.9
	√		90.13	90.64	93.03	79.68	90.34	7.8	2,326,686	4.9
		√	91.72	88.96	92.61	79.09	90.30	5.6	2,395,703	5.0
√	√		91.30	88.33	93.25	80.09	89.75	6.0	772,032	1.8
√		√	89.66	89.44	92.49	79.28	89.54	5.5	752,114	1.8
	√	√	90.35	89.67	92.69	79.72	89.97	7.2	2,221,182	4.7
√	√	√	89.51	91.26	93.47	80.24	90.34	5.4	701,696	1.7

Note: √ indicates that the corresponding component is included.

**Table 8:** Comparison of detection performance and model complexity among different object detection models.

Model	P (%)	R (%)	mAP@0.5 (%)	mAP@0.5:0.95 (%)	F_1_ (%)	FLOPs (G)	Parameters	Size (MB)
Faster R-CNN	56.99	84.39	77.16	58.40	68.03	401.8	137,098,724	522.99
SSD	74.99	55.25	67.66	22.53	63.62	274.9	24,146,894	92.11
RT-DETR-L	91.74	87.17	88.73	73.81	89.40	100.6	28,453,535	56.4
YOLOv5n	90.79	88.48	92.60	78.16	89.59	5.8	2,182,639	4.5
YOLOv8n	90.93	88.64	92.38	79.10	89.75	6.8	2,685,343	5.4
YOLOv10n	91.06	87.83	92.68	78.33	89.39	6.5	2,266,143	5.5
YOLO11n	91.84	88.35	92.30	79.02	90.04	6.3	2,583,127	5.3
YOLOv12n	90.84	87.91	92.06	78.28	89.31	5.8	2,509,319	5.3
YOLOv13n	89.76	87.14	90.19	76.33	88.41	6.1	2,448,870	5.2
LSARE-Net	89.51	91.26	93.47	80.24	90.34	5.4	701,696	1.7

**Table 9:** Class-specific detection performance and error counts of LSARE-Net on the test set.

Category	P (%)	R (%)	AP@0.5 (%)	AP@0.5:0.95 (%)	TP	FP	FN
resting	95.19	96.23	97.61	79.80	970	49	38
standing	80.14	88.05	89.56	70.14	803	199	109
feeding	74.76	85.16	85.10	69.79	459	155	80
social	90.34	89.23	93.38	84.18	290	31	35
abnormal	99.53	100.00	99.50	94.15	212	1	0

**Table 10:** Test performance and model complexity of YOLO11n and LSARE-Net across six random seeds.

Seed	Model	P (%)	R (%)	mAP@0.5 (%)	mAP@0.5:0.95 (%)	F_1_ (%)	FLOPs (G)	Parameters	Size (MB)
0	YOLO11n	91.84	88.35	92.30	79.02	90.04	6.3	2,583,127	5.3
	LSARE-Net	89.51	91.26	93.47	80.24	90.34	5.4	701,696	1.7
1	YOLO11n	90.61	89.46	92.35	78.58	90.01	6.3	2,583,127	5.3
	LSARE-Net	91.32	89.53	93.24	79.98	90.40	5.4	701,696	1.7
2	YOLO11n	91.66	87.67	92.39	79.15	89.59	6.3	2,583,127	5.3
	LSARE-Net	91.67	90.85	94.13	80.40	91.23	5.4	701,696	1.7
3	YOLO11n	89.38	89.74	92.78	78.32	89.51	6.3	2,583,127	5.3
	LSARE-Net	91.31	89.09	93.77	79.38	90.16	5.4	701,696	1.7
4	YOLO11n	90.54	89.40	92.37	78.32	89.92	6.3	2,583,127	5.3
	LSARE-Net	91.39	89.38	93.38	79.83	90.36	5.4	701,696	1.7
5	YOLO11n	91.02	88.79	92.06	78.62	89.83	6.3	2,583,127	5.3
	LSARE-Net	91.74	88.17	93.48	80.10	89.90	5.4	701,696	1.7

**Table 11:** Overall and class-specific detection performance and model complexity of YOLO11n and LSARE-Net after training within the additional scene.

Model	Category	P (%)	R (%)	AP@0.5 (%)	AP@0.5:0.95 (%)	F_1_ (%)	FLOPs (G)	Parameters	Size (MB)
YOLO11n	Overall	75.96	69.30	75.46	47.90	72.41	6.3	2,582,737	5.3
YOLO11n	resting	89.69	85.11	91.45	58.20	87.34	6.3	2,582,737	5.3
YOLO11n	standing	84.70	79.11	87.57	56.42	81.81	6.3	2,582,737	5.3
YOLO11n	feeding	53.47	43.69	47.35	29.07	48.09	6.3	2,582,737	5.3
LSARE-Net	Overall	73.70	73.13	77.06	49.88	73.40	5.4	701,436	1.7
LSARE-Net	resting	84.97	85.83	90.57	57.93	85.40	5.4	701,436	1.7
LSARE-Net	standing	82.99	83.03	88.55	59.05	83.01	5.4	701,436	1.7
LSARE-Net	feeding	53.14	50.52	52.05	32.66	51.80	5.4	701,436	1.7

Note: AP values in the Overall rows represent the mean AP across the three evaluated categories.

**Table 12:** Deployment environment of NVIDIA Jetson AGX Orin.

Hardware/Software Environment	Version
Development board	NVIDIA Jetson AGX Orin
Operating system	Ubuntu 20.04
Inference backend	TensorRT
Input size	640 × 640
Python	3.11.13
PyTorch	2.2.2 + cu121
torchvision	0.17.2 + cu121
ultralytics	8.3.9

**Table 13:** Comparison of inference performance of different models on the NVIDIA Jetson AGX Orin platform.

Model	mAP@0.5 (%)	mAP@0.5:0.95 (%)	FLOPs (G)	Parameters	FPS	Size (MB)
RT-DETR-L	88.60	73.08	100.6	28,453,535	42.72	62.6
YOLOv5n	92.39	77.65	5.8	2,182,639	82.40	7.0
YOLOv8n	92.20	78.62	6.8	2,685,343	75.18	8.0
YOLOv10n	92.54	78.00	6.5	2,266,143	120.12	7.8
YOLO11n	92.48	78.27	6.3	2,583,127	70.48	8.3
YOLOv12n	92.00	77.43	5.8	2,509,319	47.97	9.1
YOLOv13n	90.16	75.96	6.1	2,448,870	55.57	11.2
LSARE-Net	93.20	79.61	5.4	701,696	62.64	5.7

**Table 14:** TensorRT engine inference latency of the compared models on the NVIDIA Jetson AGX Orin.

Model	Mean Latency (ms)	Median (ms)	P90 (ms)	P95 (ms)	P99 (ms)
RT-DETR-L	12.028	12.014	12.045	12.052	12.070
YOLOv5n	1.369	1.357	1.359	1.360	1.365
YOLOv8n	1.493	1.479	1.482	1.483	1.487
YOLOv10n	1.661	1.646	1.648	1.649	2.005
YOLO11n	1.732	1.619	1.625	2.701	4.412
YOLOv12n	2.533	2.496	2.500	2.503	3.895
YOLOv13n	3.190	3.185	3.189	3.191	3.198
LSARE-Net	2.445	2.440	2.446	2.448	2.457

Note: All latency values are reported in milliseconds; lower values indicate faster execution. P90, P95, and P99 denote the 90th, 95th, and 99th percentiles of the 500 timed iterations, respectively.

## Data Availability

The data presented in this study are available on request from the corresponding author.

## Supplementary Materials

The following supporting information can be downloaded at: https://www.mdpi.com/article/10.3390/ani16193094/s1.
