# Supplementary material for: LSARE-Net: Target-Guided Augmentation and Lightweight Edge Detection for Real-Time Behavior Monitoring of Captive Juvenile Sika Deer
Source: Animals (Basel). 2026 Oct 1;16(19):3094. doi: 10.3390/ani16193094 (PMC13663615; doi:10.3390/ani16193094)
Supplement: Supplementary file 1 [file animals-16-03094-s001.zip › Supplementary_File_S2.pdf]

Supplementary File S2

S2.1. Scope and implementation

This supplement describes the implementation and training configurations of YOLOv5n, YOLOv8n, YOLOv10n, YOLO11n, YOLOv12n, YOLOv13n, and LSARE-Net used in the main comparison. The local experimental project was based on the Ultralytics repository (https://github.com/ultralytics/ultralytics), using framework version 8.3.9 for all seven training runs. All seven models were implemented within this project using the architecture configurations listed in Table S2.1.

Table S2.1. Architecture configurations of the seven models.

**Model**	**Configuration**	**Nano-scale settings[1]**	**Principal modules**
YOLOv5n	yolov5.yaml	0.33 / 0.25 / 1024	C3, SPPF, Detect
YOLOv8n	yolov8.yaml	0.33 / 0.25 / 1024	C2f, SPPF, Detect
YOLOv10n	yolov10n.yaml	0.33 / 0.25 / 1024	SCDown, PSA, C2fCIB, v10Detect
YOLO11n	yolo11.yaml	0.50 / 0.25 / 1024	C3k2, SPPF, C2PSA, Detect
YOLOv12n	yolo12.yaml	0.50 / 0.25 / 1024	A2C2f, C3k2, Detect
YOLOv13n	yolo13.yaml	0.50 / 0.25 / 1024	DSC3k2, A2C2f, HyperACE, FullPAD_Tunnel, Detect
LSARE-Net	Custom configuration; Supplementary File S1	0.50 / 0.25 / 1024	LSGFM, Pzconv, ASRR, EGDetect

[1] Values represent the depth multiplier, width multiplier, and maximum-channel setting, respectively. The complete LSARE-Net architecture is provided in Supplementary File S1.

S2.2. Dataset configuration

All seven models used the same main-dataset split. The training, validation, and test subsets contained 2,063, 2,358, and 1,179 original images, respectively. Offline augmentation was applied only to the training subset, increasing it to 8,252 images. Validation and test images were not augmented.

The dataset configuration specified five classes: social (0), abnormal (1), resting (2), feeding (3), and standing (4). The abnormal category refers specifically to visible abnormal recumbency and is not a diagnostic label.

S2.3. Training configuration

All seven models were trained from scratch under a common prespecified configuration, without model-specific hyperparameter tuning. Table S2.2 reports the shared training settings.

Table S2.2. Shared training settings.

**Parameter**	**Value**
Maximum training epochs	300
Early-stopping patience	100 epochs
Input image-size setting	640
Batch size	16
Data-loader workers	4

Parameter	Value
Optimizer	SGD
Initial learning rate	0.01
Final learning-rate factor	0.01
Momentum	0.937
Nesterov momentum	Enabled
Weight decay	0.0005
Nominal batch size	64
Warmup epochs	3.0
Warmup momentum	0.8
Warmup bias learning rate	0.1
Automatic mixed precision	Enabled
Image caching	RAM
Random seed	0
Deterministic execution setting	Enabled
Rectangular training	Disabled
Multi-scale training	Disabled
Cosine learning-rate schedule	Disabled
Mosaic closure	Final 10 epochs
Validation during training	Enabled

The online augmentation settings were hue, saturation, and value parameters of 0.015, 0.7, and 0.4, respectively; translation of 0.1; scale of 0.5; horizontal-flip probability of 0.5; and mosaic probability of 1.0. Rotation, shear, perspective, vertical flipping, mixup, and copy-paste were configured as zero. These online settings are distinct from the offline augmentation procedures described in Section 2.5 of the manuscript.

S2.4. Completed epochs and checkpoint selection

All seven models completed 300 training epochs. For each model, the best.pt checkpoint selected according to the training framework's validation fitness criterion was used for test-set evaluation. Test performance was not used to select checkpoints.

Table S2.3. Training histories and evaluation checkpoints.

Model	Completed epochs	Test checkpoint
YOLOv5n	300	best.pt
YOLOv8n	300	best.pt
YOLOv10n	300	best.pt
YOLO11n	300	best.pt
YOLOv12n	300	best.pt

Model	Completed epochs	Test checkpoint
YOLOv13n	300	best.pt
LSARE-Net	300	best.pt

S2.5. Evaluation settings and preprocessing

The common evaluation script loaded each model's best.pt checkpoint and explicitly specified the test subset, an input image-size setting of 640, and a batch size of 16. Confidence, IoU, rectangular batching, and numerical precision settings were not explicitly overridden in that script and were resolved by the evaluation framework.

Training-time data augmentation was disabled during test-set evaluation. Images were loaded in BGR format using OpenCV and resized using bilinear interpolation to a longest-side length of 640 pixels while preserving their aspect ratio. Subsequent letterbox processing used the rectangular batch target shapes and applied centered constant padding with a value of 114 in each color channel. Additional upscaling was disabled within the letterbox step. Images were converted from BGR to RGB and from HWC to CHW layout, converted to PyTorch tensors, and normalized by dividing pixel values by 255 before inference.

The evaluation script reported class-specific precision, recall, F1, AP at IoU 0.5 and 0.75, and mean AP over IoU thresholds from 0.5 to 0.95. Overall F1 was calculated as the arithmetic mean of the returned class-specific F1 values.

S2.6. Interpretation of the comparison

The experiments assessed model performance under a common training protocol without allocating additional hyperparameter tuning to LSARE-Net. The shared data split, batch size, maximum epoch budget, and validation-based checkpoint-selection procedure provided a controlled basis for comparison.

Identical hyperparameters do not necessarily provide equally favorable convergence across architectures, and equal epoch budgets do not imply equal computational expenditure. The results therefore characterize performance under the reported protocol rather than the individually optimized performance of each architecture.
